# SH3GLB2/endophilin B2 regulates lung homeostasis and recovery from severe influenza A virus infection

**DOI:** 10.1038/s41598-017-07724-5

**Published:** 2017-08-04

**Authors:** Kristin K. Fino, Linlin Yang, Patricia Silveyra, Sanmei Hu, Todd M. Umstead, Susan DiAngelo, E. Scott Halstead, Timothy K. Cooper, Thomas Abraham, Yoshinori Takahashi, Zhixiang Zhou, Hong Gang Wang, Zissis C. Chroneos

**Affiliations:** 1 Department of Pediatrics, Pulmonary Immunology and Physiology Laboratory, Pennsylvania State University College of Medicine, Pennsylvania, USA; 2 Department of Pediatrics, Hematology Oncology, Pennsylvania State University College of Medicine, Pennsylvania, USA; 3 Department of Pediatrics, Critical Care Medicine, Pennsylvania State University College of Medicine, Pennsylvania, USA; 4 Department of Biochemistry and Molecular Biology, Pennsylvania State University College of Medicine, Pennsylvania, USA; 5 Children’s Hospital, Penn State Health Milton S. Hershey Medical Center, Pennsylvania, USA; 6 Department of Comparative Medicine, Pennsylvania State University College of Medicine, Pennsylvania, USA; 7 Department Pathology, Pennsylvania State University College of Medicine, Pennsylvania, USA; 8 Department of Neural and Behavioral Sciences, and the Microscopy Imaging Facility, Pennsylvania, USA; 9 The College of Life Science and Bioengineering, Beijing University of Technology, Beijing, China; 10 Department of Pharmacology, Pennsylvania State University College of Medicine, Pennsylvania, USA; 11 Department of Microbiology and Immunology, Pennsylvania State University College of Medicine, Pennsylvania, USA

## Abstract

New influenza A viruses that emerge frequently elicit composite inflammatory responses to both infection and structural damage of alveolar-capillary barrier cells that hinders regeneration of respiratory function. The host factors that relinquish restoration of lung health to enduring lung injury are insufficiently understood. Here, we investigated the role of endophilin B2 (B2) in susceptibility to severe influenza infection. WT and B2-deficient mice were infected with H1N1 PR8 by intranasal administration and course of influenza pneumonia, inflammatory, and tissue responses were monitored over time. Disruption of B2 enhanced recovery from severe influenza infection as indicated by swift body weight recovery and significantly better survival of endophilin B2-deficient mice compared to WT mice. Compared to WT mice, the B2-deficient lungs exhibited induction of genes that express surfactant proteins, ABCA3, GM-CSF, podoplanin, and caveolin mRNA after 7 days, temporal induction of CCAAT/enhancer binding protein CEBPα, β, and δ mRNAs 3–14 days after infection, and differences in alveolar extracellular matrix integrity and respiratory mechanics. Flow cytometry and gene expression studies demonstrated robust recovery of alveolar macrophages and recruitment of CD4+ lymphocytes in B2-deficient lungs. Targeting of endophilin B2 alleviates adverse effects of IAV infection on respiratory and immune cells enabling restoration of alveolar homeostasis.

## Introduction

Pathogenic influenza A virus (IAV) strains that arise frequently by mutation or re-assortment of the viral genome have persistently caused seasonal epidemics, regional zoonotic infections, and periodic global pandemics with high morbidity and mortality for centuries^1, 2^. IAV causes productive infection in airway epithelial cells enabling the spread of infection to the lower respiratory tract, disruption of surfactant metabolism, and loss of epithelial barrier integrity^3–5^; restrictive infection that primes activation of capillary endothelial cells^6^; and abortive infection in alveolar macrophages and dendritic cells that contributes to induction of innate and adaptive immunity^7, 8^. The variable life cycle and genetic and antigenic diversity of IAV strains may determine IAV pathogenicity in different host cells^9^. The respiratory illness caused by severe IAV infection is a poorly resolving post-viral pneumonitis that can progress to acute respiratory distress syndrome (ARDS) and may be fatal depending on the extend of lung injury^10, 11^. Pathogenic IAV strains disrupt endothelial and epithelial tight junctions^4^, enhancing microvascular permeability and leakage of fluid into air sacs, and perturb surfactant lipid and protein metabolism^3^ by alveolar type II epithelial cells increasing surface tension at the air-liquid interface. In addition to functional disruption of the respiratory barrier, IAV infection elicits apoptosis and ER stress that may further aggravate tissue damage and inflammation^12–14^. The incipient edema, respiratory imbalance between air and fluid-filled alveoli, and the collateral immune response to the viral infection exacerbate respiratory injury that manifest as excessive production of inflammatory mediators, diffuse alveolar damage and hypoxia in terminal ARDS^11^. Thus, a better understanding of the host factors that contribute to IAV pathogenicity is crucial for the development of therapies that prevent pneumonia and intractable lung injury from IAV infection.

Endophilin B2 is a member of the endophilin family of proteins containing an amino-terminal Bin-Amphiphysin-Rvs (N-BAR) domain^15, 16^. BAR domains are involved in protein-protein dimerization, membrane binding, and curvature formation and sensing^16^. B2 dimerizes with its better known paralog endophilin B1(B1), which promotes mitochondrial apoptosis^16, 17^, autophagosome formation^18^, endocytic degradation of growth factor receptors^19^, and adipose tissue lipid metabolism and insulin resistance^20^. The B1/B2 heterodimer has been found to be indispensable for degradation of the inner mitochondrial membrane and mitophagy for autophagy-dependent clearance of damaged mitochondria^21^. Interestingly, B2 binding to plectin 1 modulates nuclear cytoskeletal positioning and mechano-transduction^22, 23^.

The present study examines the role of B2 expression in pathogenesis of IAV infection using transgenic mice and is based on our recent findings that loss of B2 delays formation of autophagosomes, endosomal acidification, and endosomal trafficking of IAV *in vitro* ^24^. The results identify endophilin B2 as a novel host factor that promotes IAV pathogenicity *in vivo*.

## Materials and Methods

### Mice

Endophilin B2-deficient *Sh3glb2* ^*Gt(OST224737)Lex*^ (B2−/−) mice were generated using 129/SvEV ES cells and C57BL/6 J recipients as recently described^24^, backcrossed for 10 generations to the C57BL/6 J background, and propagated by brother to sister mating^24^. Control WT C57BL/6 J purchased from The Jackson Laboratory (stock number 000664, Bar Harbor, ME) were bred and maintained locally under identical husbandry conditions. Age matched female mice were used in all experiments. All animal procedures were reviewed and approved by the Institutional Animal Care and Use Committee at Pennsylvania State University College of Medicine in accordance to the regulations of the 8^th^ edition of the GUIDE for the Care and Use of Laboratory Animals adopted in the 2015 Public Health Service Policy on Humane Care and Use of Laboratory Animals by the National Institutes of Health Office of Laboratory Animal Welfare, USA.

### Virus preparation and infections

Influenza strain A/8/Puerto Rico/34 (PR8) was grown in the chorioallantoic fluid of ten day old chicken eggs and purified by discontinuous sucrose gradient centrifugation as previously described^25^. A dose of 1000 fluorescent focus counts (ffc) of IAV H1N1 PR8 in 40 μL of Dulbecco’s Phosphate Buffered Saline (DPBS) was instilled intranasally under ketamine/xylazine anesthesia. Mice were monitored for body weight loss and survival as described previously^25^.

### Lung and BAL Harvesting

Anesthetized mice were intra-tracheally cannulated with Intramedic™ (BD Bioscience, Franklin Lakes, NJ) polyethylene tubing (ID: 0.58 mm, OD: 0.965 mm) and BAL cells recovered using a total of 2.5 mL DPBS with 1 mM EDTA as previously described (1). For isolation of lung cells, lungs were perfused with 10 mL of DPBS via the right cardiac ventricle and lavaged once with 1 mL HBSS with Ca^+2^ and Mg^+2^. An enzyme digest solution (50 μg/mL DNase I (Roche) in HBSS Ca^+2^ and Mg^+2^(HBSS, Gibco®, Grand Island, NY) and 3 mg/mL of Liberase DL (Roche, Indianapolis, IN) was instilled into the lungs intratracheally. Lungs were then excised and digested with the enzyme digest solution for 30 min at 37 °C. Lung tissue was further dispersed by repeated passage through an 18 G needle and syringe and further incubation for 15 min at 37 °C. Digestion was stopped with DPBS with 2% FBS/5 mM EDTA and digests were filtered through 40 μm mesh (BD Bioscience) to yield a single cell suspension. Red blood cells (RBCs) were lysed using RBC lysis buffer (eBioscience, San Diego, CA) and the lung cells used for flow cytometry together with cells obtained by BAL.

### Flow cytometric cell surface staining

Cell suspensions were blocked with DPBS/2% FBS containing 5 μg/mL Fc Block (eBioscience) for 1 hr at 4 °C. Blocked cells were stained fluorochrome conjugated monoclonal antibodies in HBSS/3% FBS/0.02% sodium azide, obtained from either BD Bioscience - SIGLECF (E50-2440), CD64 (X54-5/7.1), CD11c (HL3), CD4 (RM4-5), CD8 (53-6.7); or eBioscience - CD45 eFluor 605 (30-F11), MHC class II (I-A/I-E; M5/114.15.2), F4/80 (BM8), Ly-6C (HK1.4); or Biolegend – Ly-6G (1A8), CD11b (M1/70); Biolegend-CD90.2 (clone 30-H12); BB15 CD8, e650 CD4. Cells were washed in DPBS alone and incubated with a Fixable Viability Dye eFluor® 780 (eBioscience) for 20 min at 4 °C to discriminate non-viable cells. Data were collected using a BD LSR flow cytometer and analyzed using FlowJo version 9.8.5 (Treestar, Mountain View, CA) using the gating strategy shown on Supplementary Figure S1. Flow cytometric data were collected using an LSR II (Becton Dickinson) instrument in the Penn State College of Medicine Flow Cytometry Core Facility.

### Western blot analysis and densitometry

Mouse lung lavage was concentrated 10-fold by lyophilization and resuspended in SDS-PAGE reducing buffer. Proteins were separated on 4–15% SDS-PAGE gradient gels and electrophoretically transferred to PVDF membrane (Immobilon-P, Cat# ISE00010, EMD Millipore, Billerica, MA). The blots were then blocked in Tris-buffered saline, pH 7.5, supplemented with 0.2% Tween 20, and 5% non-fat dry milk. Blots were probed with anti-SP-B antibodies (Seven Hills Bioreagents, Cincinnati, OH) followed by HRP conjugated anti-rabbit antibodies (Bio-Rad Laboratories, Inc., Hercules, CA). Bound antibodies were visualized by ECL (PerkinElmer, NEL104001EA). Relative band intensity was determined by densitometry using a GS-800 Calibrated Densitometer (Bio-Rad Laboratories, Inc.) and Quantity One software (Bio-Rad Laboratories, Inc.).

### Cytokine measurements in BAL

Albumin in BAL was measured using an ELISA kit according to the manufacturer’s recommendations. (Cat. No. E90-134, Bethyl Laboratories, Inc., Montgomery, TX).

### Quantitation of viral and host genes

Lungs from vehicle treated and IAV infected mice were weighed and homogenized on ice in Trizol® (ThermoFisher Scientific, Waltham MA) using a Polytron® homogenizer for 15–30 secs and stored frozen at −80 °C until extraction of RNA. RNA was extracted with chloroform, precipitated with isopropanol, and cleaned using the QIAGEN RNeasy Kit. For quantitation of viral titer, cDNA was synthesized with the High Capacity cDNA Reverse Transcription Kit (Invitrogen) using the IAV MP primer 5′ TCT AAC CGA GGT CGA AAC GTA 3′ for IAV or oligo DTs following the manufacturer’s protocol. The cDNA was diluted five-fold prior and PCR amplified using the TaqMan Fast Universal PCR Master Mix (ThermoFisher. The A/8/Puerto Rico/34 M1 gene was amplified using the following primers: sense: 5′-AAG ACC AAT CCT GTC ACC TCTG A-3′ and antisense: 5′-CAA AGC GTC TAC GCT GCA GTC -3′, 900 nM each and the 200 nM probe sequence: 5′-/56-FAM/ TTT GTG TTC ACG CTC ACC GT/36-TAMSp/-3′. The PCR reactions were run in triplicate on a Quant Studio 12KFlex (Applied Biosystems) using 384-well optical plates at 50 °C for 2 min, 95 °C for 10 min, and 45 cycles of 95 °C for 15 seconds and 60 °C for 1 min (Genomics Sciences Core Facility, Pennsylvania State University College of Medicine). Viral copy load was quantified against a standard curve of the viral M1 RNA segment. Mouse genes (*Ifnl2*: 042044158, *Ifnl3*: Mm00663660, *Ifng:* Mm04204158; *Sftpa1*: Mm00499170, *Sftpb*: Mm00455673, *Sftpc*: Mm00488145, *Sftpd*: Mm00486060, *Abca3*: Mm00550501, *Pdpn*: Mm00494176, *Cav1*: Mm00483057, *Csf2*: Mm01290062, *Nos2*: Mm00440502, *Cebpa*: Mm00514283, *Cebpb*: Mm00853434, *Cebpd*: Mm00786711, *Arg*1: Mm00475988, *Siglecf*: Mm00523987, *Itgax*: Mm00498701, *Itgam*: Mm00434455, *Spi1*: Mm00488140) were quantitated qRT-PCR using TaqMan assays (ThermoFisher) and normalized to 18S rRNA. Data were expressed normalized relative quantity (RQ) compared to a WT vehicle control.

### Respiratory mechanics

Parameters of lung function were measured using the forced oscillation technique (FOT) and a computer-controlled flexiVent FX ventilator (SCIREQ, Montreal, Canada) as previously described by Martin *et al*.^26^. Briefly, mice were anesthetized with a mixture of ketamine and xylazine and the trachea cannulated with a 19 G cannula. Mice were then connected to the flexiVent via the cannula and ventilation started using oxygen containing 1% Isoflurane at a respiratory rate of 150BPM. Vecuronium bromide, a non-polarizing paralytic, was also administered at this time to block respiratory movements. After 5 min of ventilation, manual Deep Inflation and manual PV Loop (PVs-P) scans were performed to collect baseline parameters before starting the inhaled Methacholine dose response scans. Methacholine (acetyl-β-methylcholine chloride, Sigma-Aldrich, St Louis, MO) was freshly prepared in DPBS at doses of 0, 1.56, 3.13, 6.25, 12.5, 25 and 50 mg/mL immediately prior to the start of each experiment and administered using the flexiVent Aeroneb fine particle nebulizer. The script used for the inhaled dose response included two Deep Inflation scans followed by twelve repeats of alternating SnapShot (sinusoidal – single frequency forced oscillation waveform) and Primewave (broadband – multi-frequency forced oscillation waveform) scans for each dose of methacholine including a baseline (no DPBS, no methacholine) dose. Data were then analyzed using flexiWare software (SCIREQ) and exported to Excel for further analyses.

### Histopathology

Mouse lungs were inflation fixed for 1 min in DPBS/4% paraformaldehyde and then overnight enbloc by immersion in fixative. Lung lobes were separated, cut transversely, paraffin embedded, and stained with either H&E to visualize histopathology, or picrosirius red to stain for collagen. All tissues were examined by an ACVP diplomate pathologist (TKC) blinded to treatment and genotype. All microscopic images were obtained with an Olympus BX51 microscope and DP71 digital camera using cellSens Standard 1.12 imaging software (Olympus America, Center Valley, PA). Peribronchial lymphocytic cuffing was scored 0–4 according to the following criteria: 0: few or no lymphocytes around the major airways, bronchioles, or blood vessels; 1: Scant infiltration with very low numbers of lymphocytes, plasma cells and macrophages; 2: mild infiltration with extension of the infiltrates along the major airways, bronchioles, or blood vessel; 3: moderate increase in the numbers of lymphoid cells in any location; 4: almost complete cuffing of bronchi, bronchioles, and blood vessels by lymphoid cells. Picrosirius red stained sections were visualized by optical birefringence microscopy. Five non-overlapping fields/section containing terminal bronchioles, alveolar ducts, atria and alveoli but not larger airways, large blood vessels, or pleura were imaged at 200x magnification. Collagen birefringence intensity was quantitated using Adobe Photoshop CS3.

### Multiphoton microscopy tissue processing

Mouse lungs were inflation fixed for 1 min in 4% paraformaldehyde in DPBS and then removed and immersed in fixative for 1 hour. Lungs were rinsed and washed with DPBS for two hours before incubating overnight in 30% sucrose, 0.02% sodium azide in DPBS. The following day lungs were incubated in 50% OCT, 15% sucrose in DPBS for 2 hours. Individual lobes for each lung were then dissected out and arranged in cryomolds and embedded in OCT. Cryomolds were placed on ice for 1 hour, frozen on dry ice, and stored at −80 °C before processing for imaging.

### Multiphoton and Harmonic Generation Imaging methods

The OCT embedded frozen whole lung cross-sections (~2 mm thick) were thawed at room temperature and immobilized on a flat surface inside a small dish. These unstained tissues were washed several times and then immersed in DPBS. Multiphoton imaging operations were performed directly on these unstained tissue sections over at least three different alveolar regions. The lung alveolar wall matrix, specifically fibrillar collagens and elastin fibers, was identified in 3-dimentional space using second harmonic generation (SHG) and multiphoton excitation fluorescence (MPEF) as described in detail previously^27, 28^. A femtosecond infrared laser source can induce harmonic generation signals from fibrillar collagens, while the same laser source can induce endogenous fluorescence from elastin fibers, enabling direct visualization and quantitation of both fibrillar collagens and elastin structures without the use of exogenous probes, histological sectioning or staining. The immobilized whole lung cross-sections were imaged using Nikon A1 MP + Multi-Photon Microscope system (Nikon Instruments, New York)^28, 29^. The laser used for SHG as well as the fluorescence emission from elastin was a mode-locked femto-second Spectra-Physics InSight DS femtosecond single-box laser system with automated dispersion compensation tunable between 680–1300 nm (Spectra-Physics, Mountain View, CA). The power attenuated laser was directed to a Nikon scan head coupled with Nikon upright microscope system (Nikon Instruments, New York). The laser beam was then focused on the specimen through a high numerical aperture, low magnification, long working distance, dipping objective, CFI75 Apo Water 25X/1.1 LWD 2.0 mm WD specifically designed for deep tissue imaging and other *ex vivo*/*in vivo*/*in vitro* imaging. The backscattered emission from the sample was collected through the same objective lens. Nikon NIS Element Software was used for the image acquisition. Non-descanned detectors and spectral scanning mode both in the reflection geometry were used for capturing the 3D images as well as for the spectral signal measurements respectively. High-sensitivity GaAsP detectors were used for very efficient SHG and elastin signal collections. A 750 nm Dichroic was used to prevent the scattered IR laser radiation from reaching the detector. A 455 long pass dichroic beam splitter (455 DCXRU, Chroma Technology, USA) was used to separate SHG signal from the MPEF signal. A 593 nm long pass dichroic beam splitter (FF593-Di03-25 × 36, Semrock Inc, USA) was used to separate other endogenous fluorescence signal above 593 nm. SHG signal was captured using a 440/20 nm SHG band pass filter (MP 440/20, Chroma Technology). Spectral measurements to confirm the specificities of SHG and elastin signals in the lung tissues were performed using 32-channel Nikon Spectral Detector integrated with Nikon A1 MP + Multi-Photon Microscope system (Nikon Instruments, New York).

For 3D image data set acquisition, the multiphoton excitation beam (tuned to 880 nm) was first focused at the maximum signal intensity focal position within the tissue sample and the appropriate detector levels (both the gain and offset levels) were then selected to obtain the pixel intensities within range of 0-4095 (12-bit images) using a color gradient function. Later on, the beginning and end of the 3D stack (i.e. the top and the bottom optical sections) were set based on the signal level degradation (~600 μm). A series of 2D Images for a selected 3D stack volume were then acquired at scan speed i.e. 4 sec per 1024 × 1024 pixels. The 3D stack images with optical section thickness (z-axis) of approximately 1.0 μm were captured from tissue volumes. The spectral unmixing was performed to extract elastin signal from other endogenous fluorescence signal using measured elastin emission signal. The unmixing algorithm is based on the assumption that the total emission (*S*) of every channel (λ) is expressed as a linear combination of the contributing endogenous fluorescence emissions.

### 3D image reconstruction and analysis

For each tissue volume reported here, z-section images were compiled and finally the 3-dimensional image restoration was performed using VOLOCITY (Perkin Elmer, UK). We calculated the mean intensities of the 3D image data set occupied by a collagen and elastin structures using the following procedure. The mean intensity estimation was performed on the 3-dimensional MPEF/SHG mage data sets recorded from three different areas of lung samples. The depth of the tissue subjected to the analysis ~600 μm thickness. We applied a noise removal filter whose kernel size of 3 × 3 to remove noise, and the lower threshold level in the histogram was set to exclude all possible background voxel values. Sum of all the voxels above this threshold level is determined to be total elastin or collagen structures. We then systematically compared 3D image volume of lung tissues generated using similar imaging conditions.

### Statistics

Graphs, survival curves, and statistical data were generated using Prism 6 for Windows (GraphPad, La Jolla, CA). Groups were compared using the multiple t-test and p values calculated using the Holm-Sidak Method. Differences were considered significant at p < 0.05.

## Results

### Disruption of endophilin B2 enhances recovery and survival from severe influenza A virus infection

**Figure 1:**
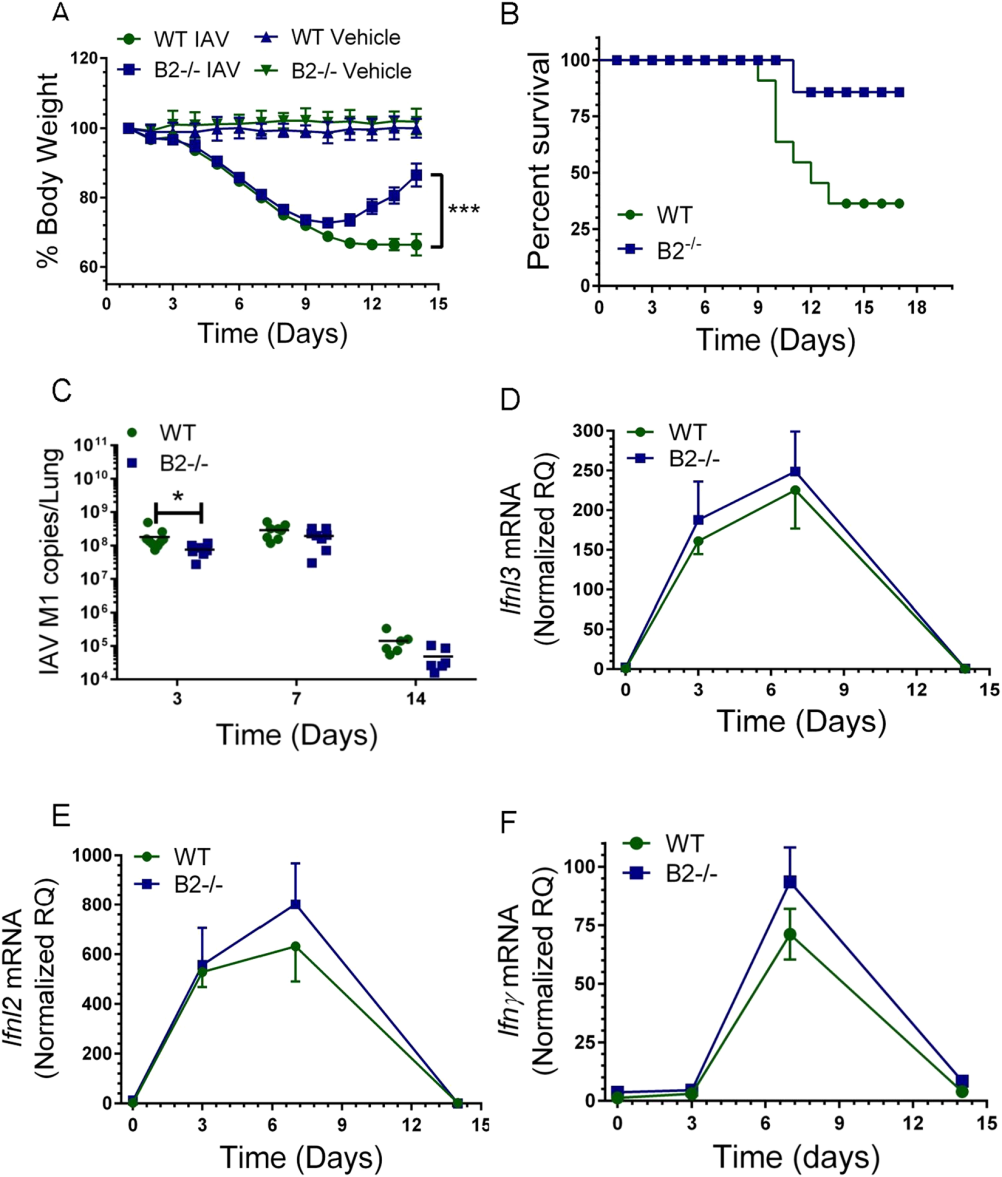
Endophilin B2 prevents recovery from severe influenza infection. Susceptibility and antiviral responses to influenza A virus (IAV) infection in WT and B2−/− mice. WT and B2−/− mice were infected with 1000 ffc of IAV H1N1 strain PR8 by intranasal administration. Uninfected WT and B2−/− mice received DPBS vehicle. (**A**) Body weight was recorded daily over 14 days. N = 31 WT mice and n = 41, B2−/− mice from 3 independent experiments. ***p < 0.0001 B2 compared to WT. (**B**) Kaplan-Meier survival curve of WT and B2−/− mice. n = 19 WT mice and 17 B2−/− mice from 2 independent experiments *p < 0.04 B2−/− compared to WT. (**C**) The viral titers were determined as the number of IAV M1 RNA copies in total lung. *p < 0.03 at t = 3 days for WT compared to B2−/− mice (for WT n = 9, 8, and 6 and B2−/− n = 7, 8, and 6 at t = 3, 7, and 14 days). (**D–F**) Expression of *Ifnl2* (**D**), *Ifnl3* (**E**), and *Ifng* (**F**) were quantitated by qRT-PCR in total lung. Data were normalized to 18 S rRNA and expressed relative to mRNA from WT uninfected control treated with DPBS vehicle (for WT n = 4, 9, 8, and 6 and B2−/− n = 3, 7, 8, and 6 mice at t = 0, 3, 7, and 14 days, respectively; the vehicle treated mice are plotted as t = 0).

To investigate the role of endophilin B2 in IAV infection, WT and B2-deficient mice (B2−/−) were infected with 1000 ffc of the influenza H1N1 PR8 strain and severity of influenza was assessed by comparing body weight, survival and viral burden over time (Fig. 1). Compared to WT mice, disruption of endophilin B2 significantly improved body weight recovery (Fig. 1A) and survival (Fig. 1B) from IAV infection. Viral proliferation was reduced in B2−/− lungs at early stage of infection (Fig. 1C). The expression kinetics of type III interferons *Ifnl2* (Fig. 1D), *Ifnl3* (Fig. 1E) and IFNγ (Fig. 1F), however, were not different.

### Endophilin B2 modulates alveolar barrier function and responses to influenza-induced injury

**Figure 2:**
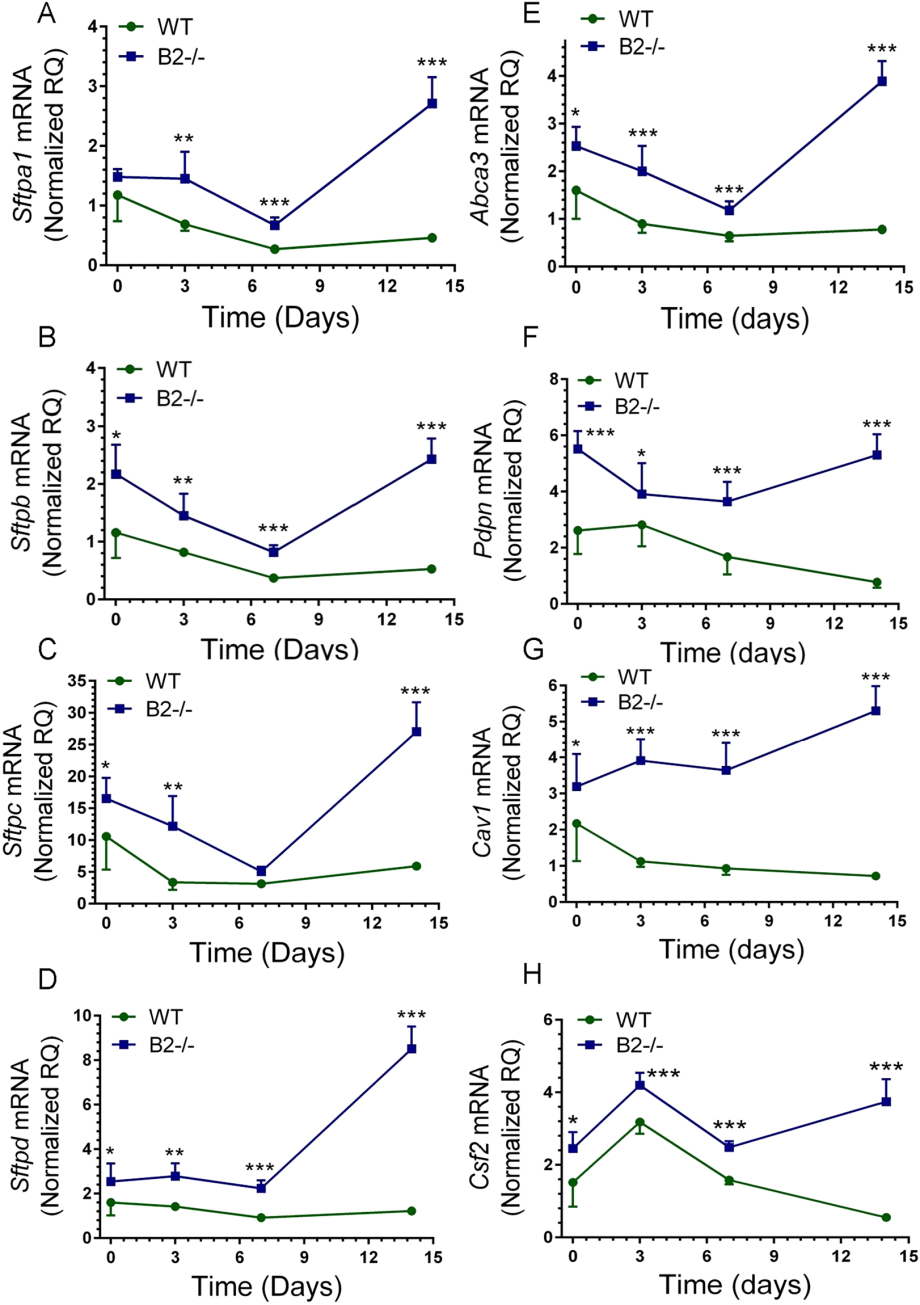
Disruption of endophilin B2 prevents suppression of alveolar epithelial and endothelial genes by IAV infection. The mRNA levels of (**A**) *Sftpa1*, (**B**) *Sftpb*, (**C**) *Sftpc*, (**D**) *Sftpd*, (**E**) *Abca3*, (**F**) *Pdpn*, (**G**) *Cav1*, and (**H**) *Csf2* in WT and B2−/− mice were quantitated by qRT-PCR in total lung. Data were normalized to 18S rRNA and expressed as relative quantity (RQ) compared to WT uninfected control treated with DPBS vehicle. ***p < 0.00001: *p < 0.006 (for WT n = 4, 9, 8, and 6 and B2−/− n = 3, 7, 8, and 6 at t = 0, 3, 7, and 14 days; the vehicle treated mice are plotted as t = 0).

Development of lung injury is critically dependent on disruption of alveolar epithelial and endothelial cells. To elucidate the role of B2, expression of key alveolar epithelial and endothelial genes was measured by qRT-PCR (Fig. 2A–H). The levels of surfactant protein A (*Sftpa1*), B (*Sftpb*), and C (*Sftpc*), and the surfactant lipid transporter *Abca3* expressed by alveolar type II epithelial cells decreased and remained depressed by ≥ 50% in WT lungs while levels of surfactant protein D (*Sftpd*) were not affected (Fig. 2A–E). *Sftpa1*, *Sftpb*, *Sftpc*, and *Abca3* also declined to 50–75% below uninfected control while *Sftpd* levels were stable for the first 7 days after infection in the lungs of B2−/− mice. In contrast to WT mice, however, there was robust recovery in expression of all five surfactant related genes between 7–14 days after infection in B2−/− mice (Fig. 2A–E). The alveolar type I epithelial cell selective podoplanin gene *Pdpn* (also known as T1α) (Fig. 2F) declined and remained depressed in WT mice, whereas *Pdpn* expression returned to pre-infection levels by 14 days in B2−/− lungs (Fig. 2F). The protein transporter caveolin 1 (*Cav1*) gene, which is highly expressed in lung microvascular endothelial cells as well as alveolar type I epithelial cells was also depressed in WT, but increased almost 2-fold between 7 and 14 days after infection in B2−/− lungs (Fig. 2G). Furthermore, expression of *Csf2* (aka GM-CSF), a central paracrine mediator of alveolar macrophage differentiation and activation^30^ expressed by alveolar type II epithelial cells increased initially but then declined below uninfected controls in WT mice (Fig. 2H). In contrast, while *Csf2* expression in B2−/− mice exhibited similar kinetics as WT initially, it remained significantly elevated in B2−/− lungs (Fig. 2H) compared to WT lungs after 7 days.

**Figure 3:**
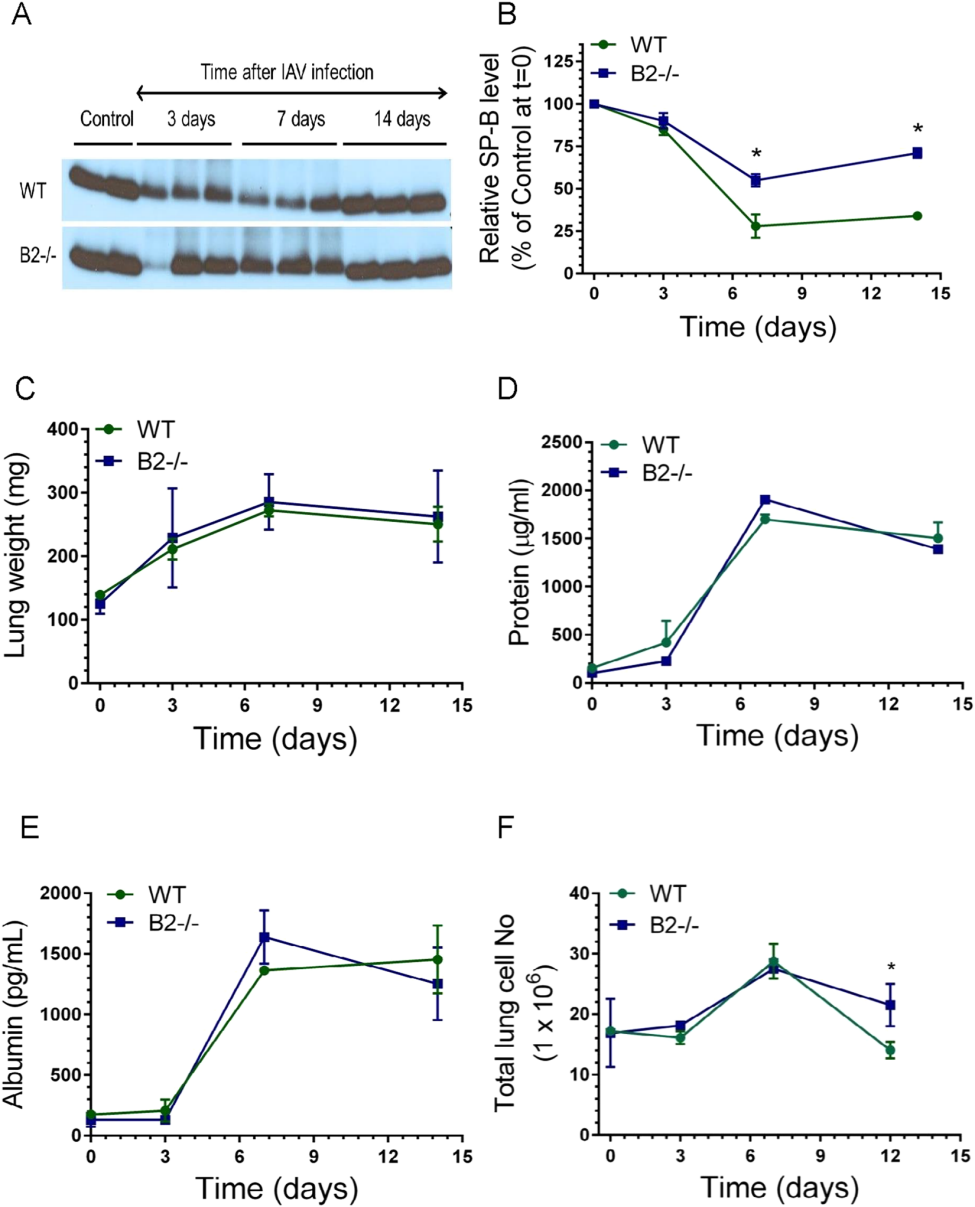
Endophilin B2 limits post-viral surfactant and lung cell depletion. (**A,B**) The levels of surfactant protein B in bronchoalveolar lavage (BAL) after infection were assessed in Western blot (**A**) by densitometry (**B**) and expressed relative to vehicle control. *p < 0.039 at t = 7 days and *p < 0.02 at t = 14 days for WT and B2−/− mice, respectively. Panel A shows representative Western blots from n = 2 independent experiments. (**C–E**) Change in wet lung weight (**C**), total BAL protein (**D**), and BAL albumin (**E**) after infection were measured by gravity, BCA, and ELISA, respectively. For WT mice, n = 5, 3, 6, and 5, and for B2−/− mice, n = 5, 6, 7, and 7 at t = 0, 3, 7, and 14 days after infection mice, respectively. (**F**) The number of live lung cells was assessed by flow cytometry. *p < 0.05 at t = 12 for B2−/− mice compared to WT. (n = 3 mice per group at t = 0, 3, and 7, and n = 12 at t = 12 from three independent determinations at 12 days after infection).

Densitometry analysis of surfactant protein B (SP-B) levels showed significantly better recovery of SP-B protein (Fig. 3A,B) consistent with the RNA data above (Fig. 2B). Wet lung weight (Fig. 3C), total protein (Fig. 3D), and albumin in BAL (Fig. 3E), however, were not different, indicating similar protein flux. Interestingly, the number of lung cells recovered by enzymatic digestion, was similar up to day 7 but was significantly higher in B2−/− mice compared to WT at 12 days (Fig. 3F).

**Figure 4:**
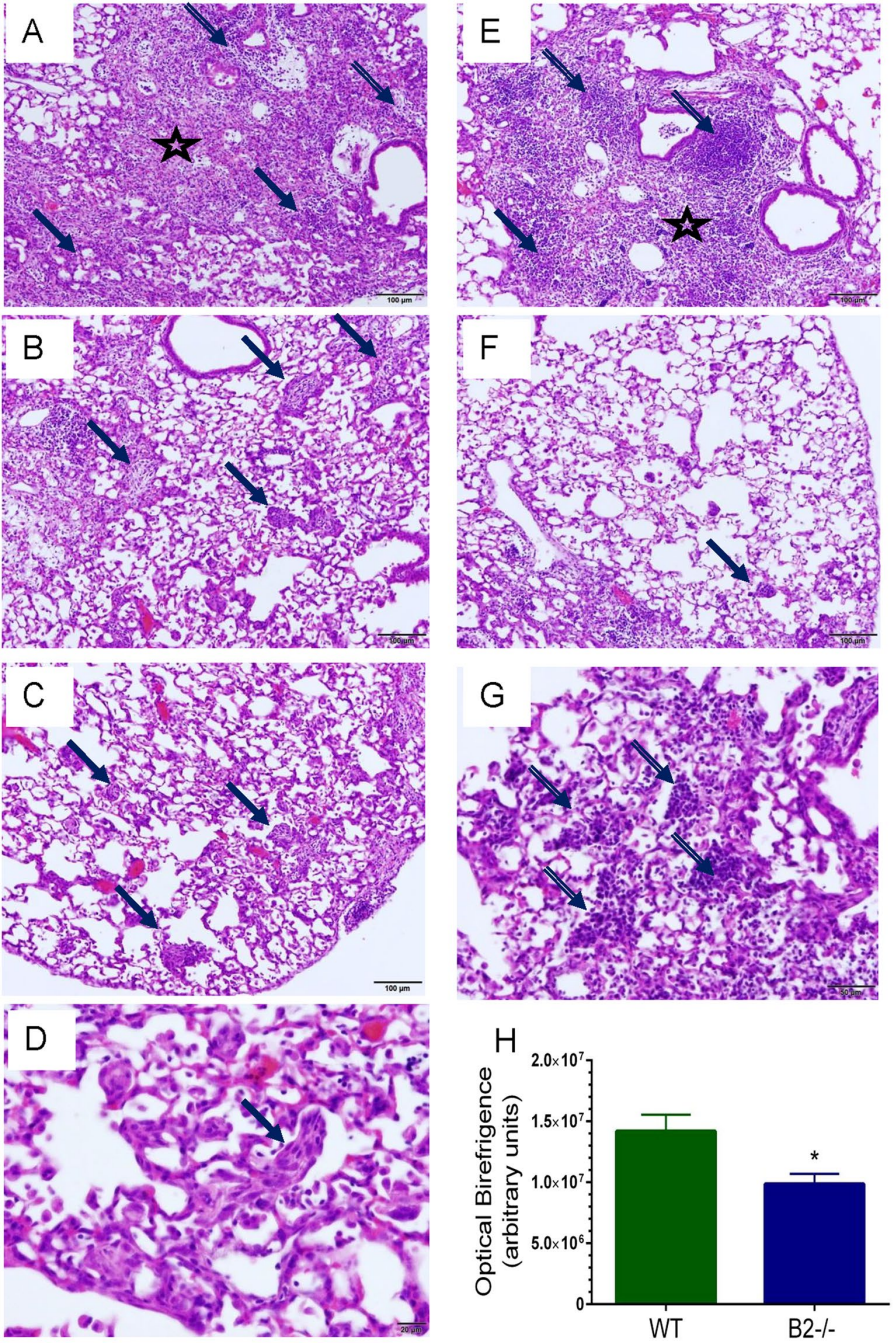
Different histopathological features in WT and B2−/− lungs after clearance of IAV infection. Histopathology was assessed 14 days after infection from two independent experiments. Images shown are representative of n = 7 WT mice and n = 5 B2−/− mice. (**A**) Fibrotic lesions (star) with areas of metaplasia (solid arrows) and disperse mononuclear infiltrates (lined arrows) consolidate the peribronchial and alveolar spaces of WT mice. (**B–D**) Cryprogenic organizing pneumonia with buds of granulation tissue in alveoli of WT mice (solid arrows). (**E**) Fibrotic lesions (star) with areas of metaplasia (solid arrow) and lymphocytic foci (lined arrows) consolidate the peribronchial and alveolar spaces of B2−/− mice. (**F**) Mild (solid arrow) to absent cryptogenic organizing pneumonia in B2 alveoli. (**G**) Alveolar lymphocytic infiltrates in B2−/− lungs. Magnification for A, B, C, E, and F: 100x; D: 600 x; G: 400x. (**H**) Comparison of collagen birefringence in WT and B2−/− lungs. Collagen was visualized picric sirius red stain. *p < 0.00046. Data shown are means ± SEM from total of 50 and 60 microscopic fields at 200x magnification in lung sections from n = 4 WT mice and n = 5 B2−/− mice, respectively.

The histological evaluation of the lungs 14 days after infection revealed differences in presentation of injury between WT (Fig. 4A–D) and B2−/− lungs (Fig. 4E–G). The lungs of both WT (Fig. 4A) and B2−/− mice (Fig. 4E) showed bronchopneumonic lesions consisting of interstitial fibrosis, squamous metaplasia, with disperse or focal lymphocytic infiltrates. Organized lymphocytic foci (Fig. 4E) were significantly more prominent in B2−/− lungs as demostrated by blind scoring of H&E stained tissue sections (Supplementary Figure S2). Cryptogenic organizing pneumonia marked by fibroblastic alveolar buds was a striking presentation of WT lung pathology (Fig. 4B–D) compared to B2 alveolar spaces that were either clear with small lesions (Fig. 4F) or infiltrated with lymphocytes (Fig. 4G). Quantitation of collagen deposition by optical birefringence showed lower deposition of collagen in broncho-alveolar septal lesions and pulmonary atria in lower airway of the B2−/− lung 14 days after infection compared to WT mice, consistent with the better recovery from lung injury in B2−/− mice (Fig. 4H and Supplementary Figures S3).

**Figure 5:**
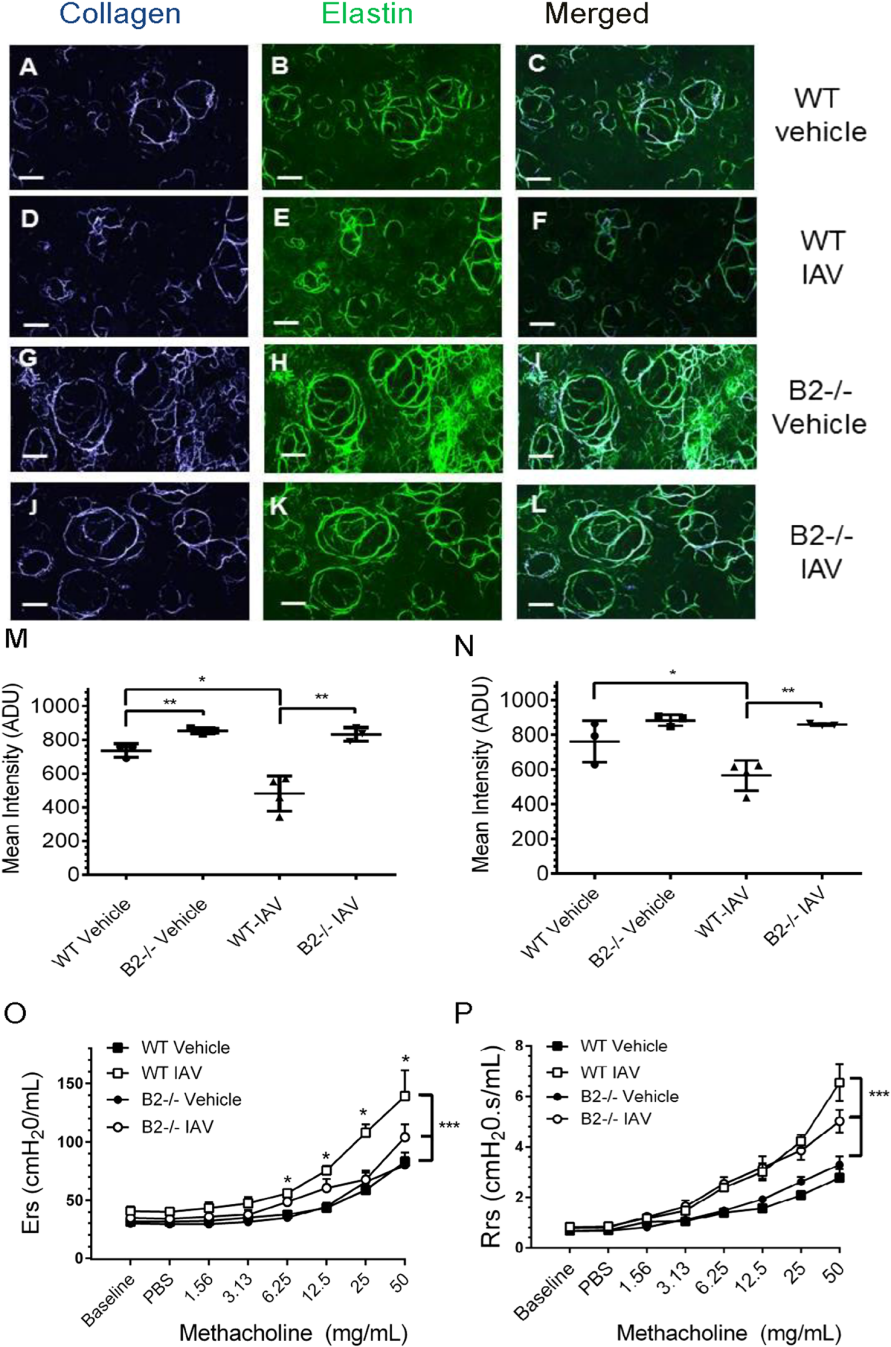
Endophilin B2 mediates disruption of alveolar wall matrix and development of respiratory disease after IAV infection. (**A–L**) Multiphoton microscopy was performed 14 days after intranasal administration of vehicle or IAV. Representative optically magnified SHG images originating from the alveolar collagen and elastin matrix are shown. The collagen (blue color) and elastic fibers (green color) are condensed around the alveolar wall in control lung tissues of both WT (**A–C**) and B2−/− mice (**G–I**) treated with vehicle as well as in B2−/− mice (**J–L**). Reduction of alveolar wall structures was evident only in WT lungs (**D–F**) 14 days after IAV infection. *Scale bar: 51* μ*m*. (**M**,**N**) Volumetric quantitation of collagen (**M**) and elastin (**N**) fibers from 3-dimensional MPEF/SHG image data sets were recorded from three different areas of WT and B2−/− lungs ~600 μm thick. The signal intensity for collagen was significantly reduced only in WT lungs 14 days after IAV infection compared to WT vehicle and IAV infected B2−/− (*p < 0.01 and **p < 0.0029). Elastin was also significantly reduced compared to WT vehicle and IAV infected B2−/− (*p < 0.043 and **p < 0.009, respectively). Collagen intensity was higher in vehicle B2−/−compared to vehicle WT (*p < 0.009). Data shown are means ± SD. (**O,P**) Respiratory elastance, Ers (**O**) and respiratory resistance, Rrs (**P**) data were acquired using a FlexiVent ventilator 14 days after administration of vehicle or IAV. *P < 0.001, and ***p < 0.0001, n = 4 per group. Data shown are means ± SD.

High resolution 3-dimentional multiphoton microscopy (MP) imaging of alveoli in freshly explanted tissue (Fig. 5A–L), however, revealed co-existing destruction of alveoli as indicated by decreased peri-alveolar collagen and elastin fibers and collapsed alveoli in WT mice 14 days after clearance of IAV infection (Fig. 5D–F) compared to vehicle control WT (Fig. 5A–C) in addition to the bronchopneumonic lesions described above (Fig. 4). Interestingly, the fibrillary collagen signal was statistically higher by 13% in B2−/− lungs compared to WT mice (Fig. 5M), suggesting basal differences in arrangement of alveolar collagen fibers, although this difference did not result in obvious alterations in basal respiratory parameters (Supplementary Figure S4). The signal intensities for fibrillar collagen (Fig. 5M) and elastin (Fig. 5N) after IAV infection were 45–50% and 25% lower in WT mice compared to vehicle treated and IAV infected WT (Fig. 5M) as well as infected and uninfected B2−/− lungs (Fig. 5N), indicating loss of alveolar structure in WT but not B2−/− mice. The loss in alveolar structure observed 14 days after infection in WT lungs is evidenced at the physiological level by the higher static compliance (Cst) measured at the end of the inspiratory curve^26^ (Supplementary Figure S4C) and decreased elastic recoil as indicated by the higher slope K of the expiratory curve^31^ (Supplementary Figure S4D), consistent with development of chronic lower airway respiratory disease after infection in WT but not B2−/− mice. Given these results, we assessed respiratory mechanics after methacholine challenge to assess disease manifestation in airway and alveolar compartments. Figure 5O shows that lack of B2 obviated obstructive disease in the distal airway as indicated by significantly lower respiratory elastance over several methacholine concentrations in B2−/− mice 14 days after infection. The increase in airway resistance in response to methacholine, however, increased similarly in WT and B2−/− mice at all but the highest concentration of methacholine (Fig. 5P), indicating similar bronchial airway disease. The vehicle treated WT and B2−/− lungs had similar responsiveness to methacholine and both significantly less responsive than infected mice (Fig. 5O–P).

### Endophilin B2 shapes immune and inflammatory cell dynamics during IAV infection and clearance

**Figure 6:**
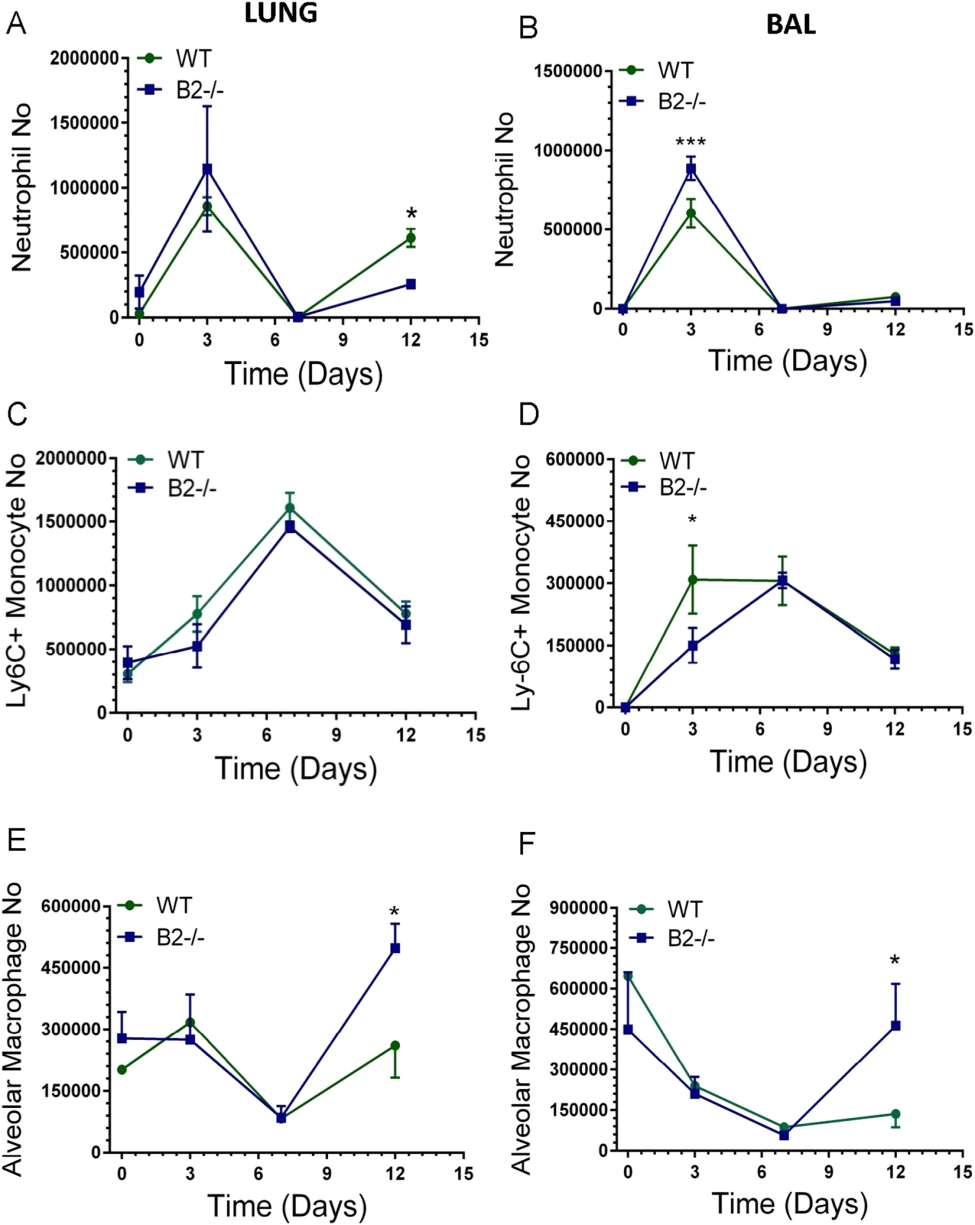
Endophilin B2 shapes inflammatory cell recruitment and suppresses recovery of AMs after IAV infection. The number of neutrophils, Ly-6C + monocytes, and AMs in lungs (**A, C, E**) and BAL (**B, D, F**), respectively, was determined by flow cytometry. Significant differences in the number of cell types between WT and B2−/− mice were calculated for (**A**) *p < 0.02 at day 12, (**B**) *p < 0.01 at day 3, (**D**) *p < 0.0002 at day 12, (**E**) *p < 0.007 at day 3, and (**F**) *p < 0.02 at day 12. (n = 3 mice per group at t = 0, 3, and 7, and n = 12 at t = 12. The data on day 12 are pooled from three independent experiments.)

**Figure 7:**
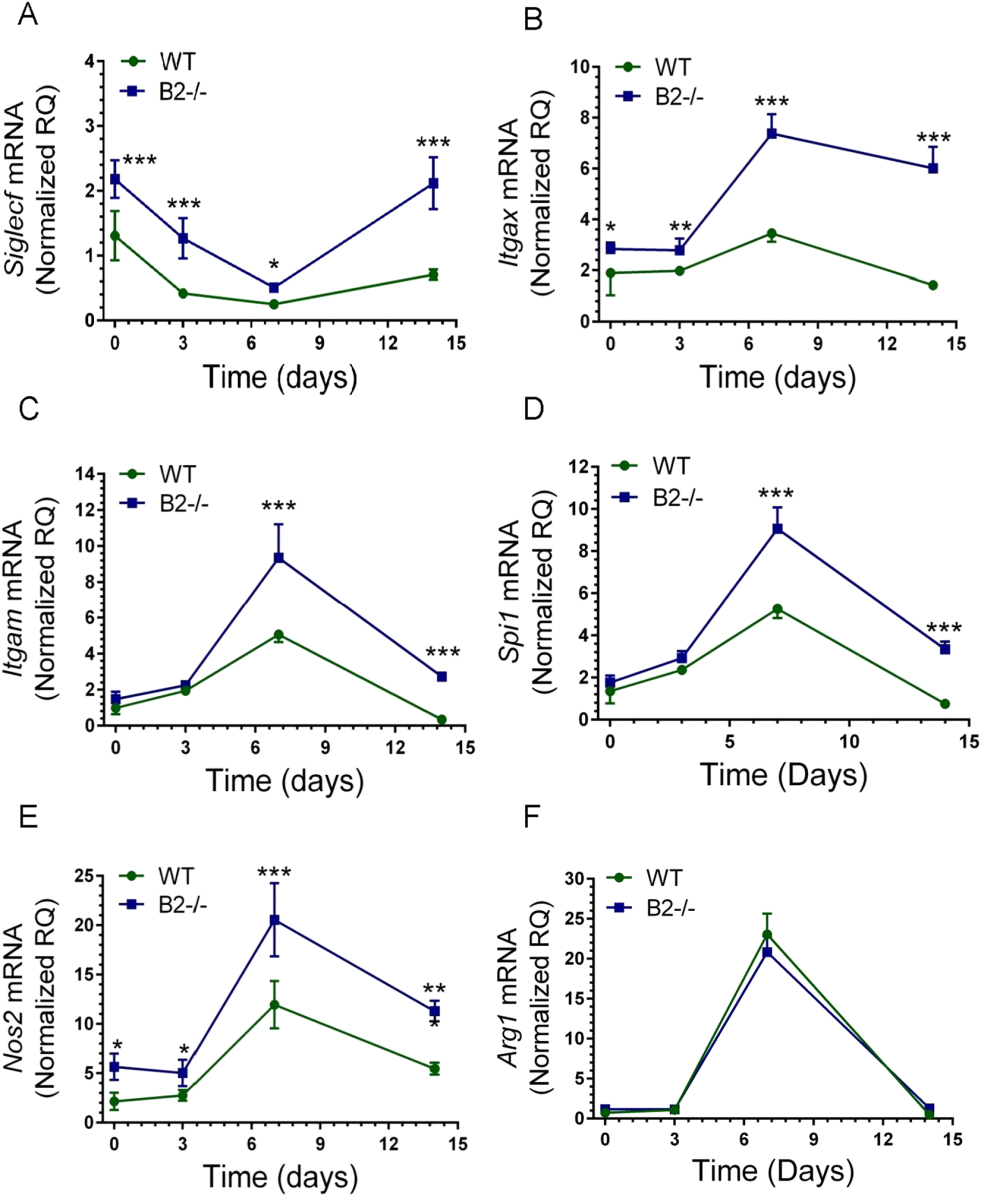
Endophilin B2 attenuates expression of macrophage differentiation and activation genes. The mRNA levels of (**A**) *Siglecf*, (**B**) *Itgax*, (**C**) *Itgam*, and (**D**) *Spi1*, (**E**) *Nos2*, and (**F**) *Arg1* in WT and B2−/− mice were quantitated by qRT-PCR in total lung. Data were normalized to 18S rRNA and expressed as relative quantity (RQ) compared to WT uninfected control treated with DPBS vehicle. ***p < 0.00001, **p < 0.001, *p < 0.05 (for WT n = 4, 9, 8, and 6 and B2−/− n = 3, 7, 8, and 6 at t = 0, 3, 7, and 14 days; the vehicle treated mice are plotted as t = 0. Data ± SEM are from 2 independent experiments).

**Figure 8:**
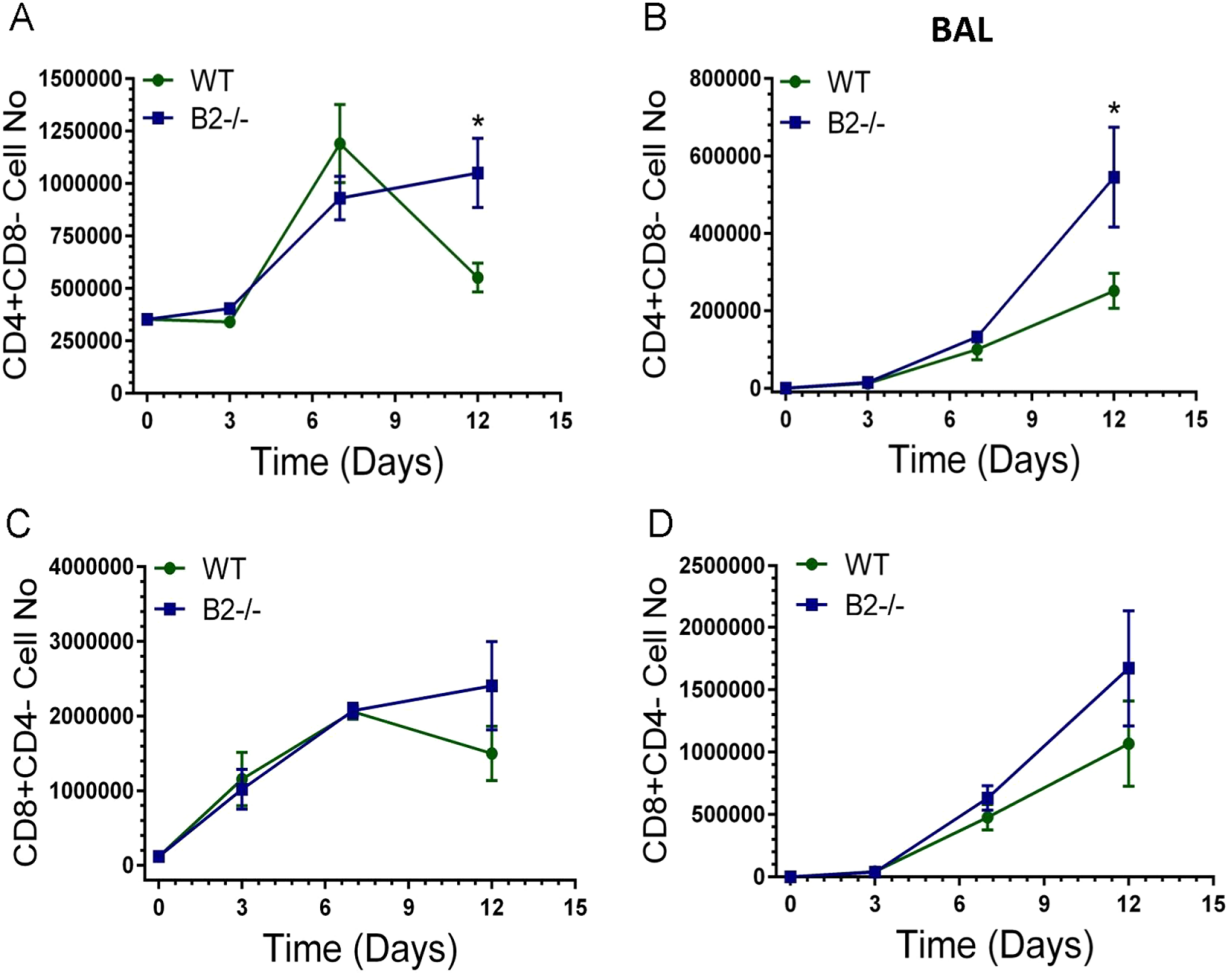
Endophilin B2 curtails contraction of CD4+ T lymphocytes recruited in lungs and BAL after IAV infection. The number of CD4+CD8- and CD8+CD4+ lymphocytes in lung (**A,C**) and BAL (**B,D**), respectively, was determined by flow cytometry. Significant differences in the number of cell types between WT and B2−/− mice were calculated for (**A**) *p < 0.002 at day 12 and (**B**) *p < 0.01 at day 12. (n = 3 mice per group at t = 0, 3, and 7, and n = 12 at t = 12. The data on day 12 are pooled from three independent experiments).

To determine whether lack of B2 alters the inflammatory response to IAV infection, multicolor flow cytometry (Supplementary Figure S1) was used to monitor alveolar macrophages and the influx of inflammatory cells in lungs and BAL (Figs 6–8). Relevant to the findings shown above in Figs 4 and 5, it has been shown that the phenotype of alveolar macrophages is shaped by the lung’s biomechanical environment^32^. Furthermore, extracellular matrix integrity influences homing and differentiation of lymphocytes in response to IAV infection^33^. Overall, the kinetics of neutrophils and Ly-6C+ monocytes was similar with some differences. The number of neutrophils in BAL of B2−/− mice was significantly higher than WT at 3 days (Fig. 6B). A second wave of neutrophils in lungs was observed in WT mice at 12 days (Fig. 6A), suggesting ongoing inflammation. The kinetics and number of Ly-6C+ monocytes (Fig. 6C) was similar, although significantly higher in BAL 3 days after infection in B2−/− mice compared to WTs (Fig. 6D). The number of eosinophils was not significantly different (Supplementary Figure S5), although also higher in B2−/− mice. IAV infection, however, caused a precipitous decline in SIGLECF + alveolar macrophages (AMs) in both lung (Fig. 6D) and BAL (Fig. 6F) of WT and B2−/− mice over the first 7 days, but AMs were then replenished in B2−/− but not WT mice by day 12. Quantitation of *Siglecf* mRNA (Fig. 7A) was consistent with the depletion and then recovery of SIGLECF + AMs (Fig. 6E,F). The robust induction of ITGAX (aka CD11c) expression after 3 days in B2−/− mice compared to WT (Fig. 7B) was consistent with differentiation and replenishment of AMs in keeping with the sustained expression of *Csf2* (Fig. 2H). The myeloid activation/differentiation markers ITGAM (Fig. 7C) and *Spi1* (aka PU.1) (Fig. 7D) increased transiently in both B2−/− and WT mice. Interestingly, expression of *Nos2* was significantly higher and remained elevated in B2−/− lungs compared to WTs (Fig. 7F), while expression of arginase 1 (*Arg1*) was similar and transient in both mouse groups (Fig. 7E), suggesting increased nitric oxide antimicrobial activity and nitric oxide signaling in B2−/− mice during recovery from IAV infection. In addition to changes in myeloid cells, disruption of B2 also altered the lymphocyte profile as indicated by the higher number of CD4 + CD8- T lymphocytes in both lungs and BAL after 7 days of infection with significant differences in CD4 + CD8- lymphocyte number measured on day 12 (Fig. 8A and B). A similar trend in the CD8 + CD4- population, however, was not statistically significant (Fig. 8C and D).

### Endophilin B2 impairs induction of CEBP transcription factors

**Figure 9:**
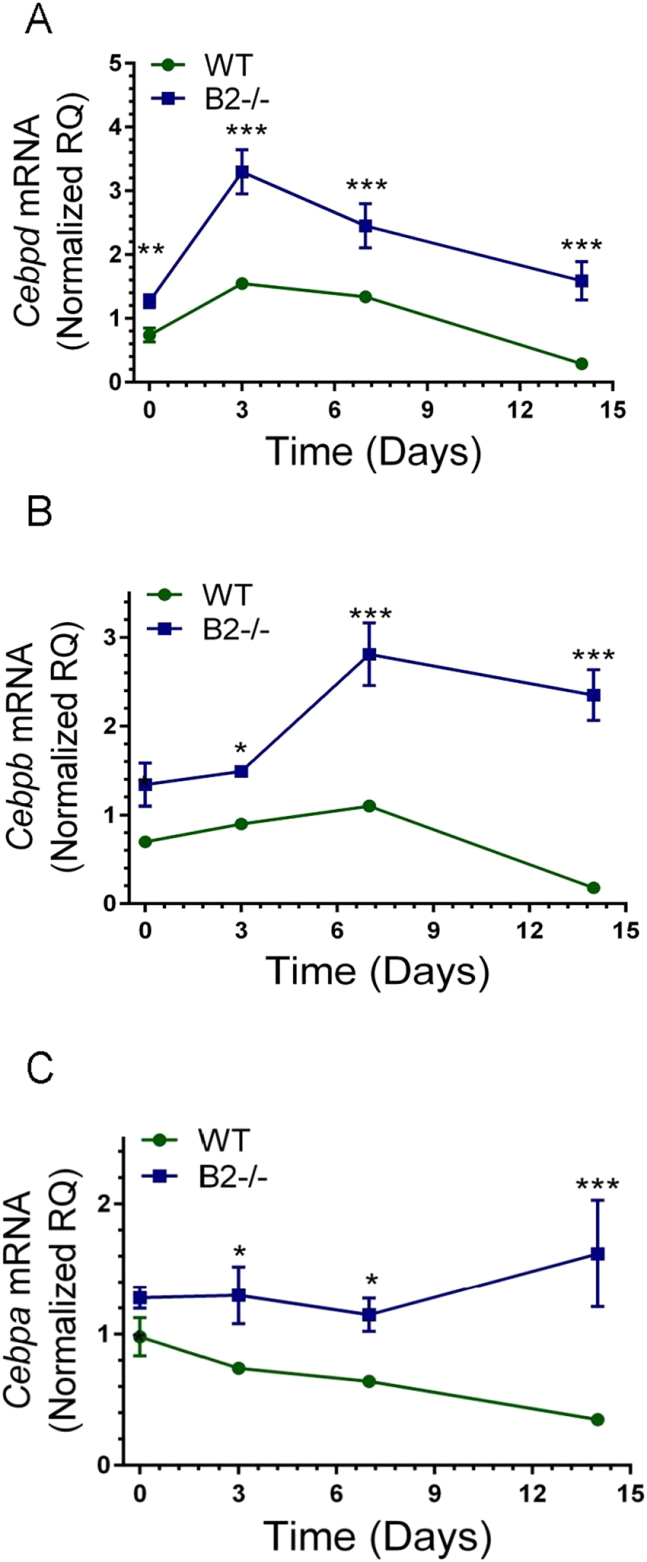
Endophilin B2 uncouples the temporal expression of CEBP transcription factors in IAV infected lugs. The expression of (**A**) *Cebpd*, (**B**) *Cebpb*, and (**C**) *Cebpa* mRNA were quantitated by qRT-PCR in total lung. Data were normalized to 18 S RNA and expressed as relative quantity (RQ) compared to WT uninfected control treated with DPBS vehicle. ***p < 0.00001, **p < 0.007, *p < 0.02 (for WT n = 4, 9, 8, and 6 and B2−/− n = 3, 7, 8, and 6 at t = 0, 3, 7, and 14 days; the vehicle treated mice are plotted as t = 0. Data ± SEM are from 2 independent experiments).

The ability of endophilin B2 to suppress gene expression in both structural and immune cells suggests a combinatorial response to IAV infection. The CCAAT/enhancer binding protein genes *Cebpd*, *Cebpb*, and *Cebpa* have crucial roles as pioneer transcription factors in both lung epithelial and immune cell regulating lung structure integrity, regeneration, differentiation, and surfactant related gene expression through interaction with tissue and cell specific transcription factors^34–39^. Disruption of *CEBPa* results in development of obstructive pulmonary disease^40^. *CEBP*β (also known as NF-IL6) mediates innate immune responses to IAV infection^41^ and vaccination^42^, and secretion of IL-6 by alveolar epithelial cells^43^; IL-6 is required to attenuate lung injury during recovery from severe IAV infection^44^. Figure 9A–C show inhibition of all three *Cebp* genes in WT mice by IAV infection. In contrast, *Cebps* were induced in a temporal fashion in B2−/− mice reaching the highest levels at 3, 7, and 14 days after infection for *Cebpd*, *Cebpb*, and *Cebpa*, respectively.

## Discussion

The present studies report, for the first time to our knowledge, that endophilin B2 suppresses recovery from severe influenza infection. IAV is capable of infecting multiple resident and incoming inflammatory cells^3–8, 45, 46^ that contribute to the complex pathogenesis of the infection. Here, we show that endophilin B2 plays a deleterious role in IAV infection by mediating persistent inhibition of surfactant protein, *Abca3*, *Pdpn*, and *Cav1* genes, which are critical for normal respiratory gas exchange, surfactant and immune homeostasis, host defense, alveolar epithelial cell differentiation, formation of caveolae, and mechano-transduction during breathing^47–52^. The restoration of genes expressed by alveolar cells in B2−/− mice reflects the increased recovery of lung cells in enzymatic digests 14 days after infection. Recovery from severe influenza infection, however, occurred despite similar transpulmonary flux of plasma proteins into the alveolar lumen. Plasma proteins can inhibit surface tension lowering properties of surfactant resulting in alveolar collapse, impaired gas exchange, and tissue injury driving development of ARDS^53, 54^. Lung protective ventilation is currently the only supportive intervention to limit the deleterious effects of edema on surfactant function^55^. It is thus plausible that correction of surfactant deficiency in B2−/− mice neutralized inhibitory and injurious effects of plasma proteins on surfactant function. The protective response to infection in B2−/− mice also was also associated with temporal deployment of CEBP transcription factors, continued expression of *Csf2*, and replenishment and differentiation of alveolar macrophages (AMs). CEBPs regulate surfactant protein gene expression and activation of macrophages and host responses to influenza infection^41–43^ while GM-CSF is critical for AM differentiation, surfactant homeostasis, and survival from lethal IAV infection^56, 57^. Furthermore, imaging and respiratory physiology studies suggest that IAV infection through B2 may alter the compartmental organization and mechanical properties of alveolar extracellular matrix in lung parenchyma. In this context, we recently reported that the ubiquitously expressed B2 delays lysosome acidification and endosomal trafficking of IAV in different cell types *in vitro* ^24^. Taken together, the present findings indicate that IAV utilizes B2 to disconnect spatiotemporal gene interactions in response to IAV infection in the lung, culminating in the development of refractory immune and structural lung injury.

Histological and physiological evidence indicated that B2 mediates destruction of alveoli secondary to viral infection that manifests in part as co-existent alveolar destruction and cryptogenic organizing pneumonia (COP). Persistence of influenza antigen on alveolar epithelial cells exacerbates immunopathology by activated CD8+ lymphocytes^58^. The presence of COP suggests that B2 limits clearance of residual viral antigen in the lower airway. It has been shown that influenza antigen persists long after viral proliferation has been terminated in the lung^59^. COP sequelae are increasingly recognized manifestations of influenza-induced pneumonitis^60–65^. Our recent studies *in vitro* also showed that loss of B2 delays formation of auto-phagolysosmes^24^ and previous studies demonstrated that suppression of basal autophagy in alveolar type II epithelial cells enhances resistance to IAV infection^66^. It is possible that evasion of B2 enhances autophagy-mediated persistence of influenza antigens on epithelial cells driving development of pneumonitis by inflammatory lymphocytes.

The remarkable recovery of alveolar-epithelial barrier genes in B2−/− infected mice also warrants deeper investigation into the role of B2 in mechano-transduction and development of mechanical injury in response to infection. Our imaging and respiratory physiology findings suggest that B2 influences alveolar collagen and elastin extracellular matrix. In this regard, it is noteworthy that extracellular matrix is critical for the homing and differentiation of CD4+ tissue resident memory lymphocytes in response to IAV infection^33, 67^. Mechanical stretch during breathing is a physiological signal for surfactant secretion^68^. Abnormal stretch can damage membrane and initiate injury and inflammatory bio-trauma^47, 69^. It is thus possible that membrane wounding that occurs during viral entry and release of virions^70, 71^ impairs epithelial membrane repair initiating inflammatory responses to physiological stress. In this context, recent studies demonstrated that B2 interacts with the N-terminal domain of the cytolinker plectin 1. This interaction affects nuclear shape and positioning, cellular signaling and chromatin methylation, and mechano-transduction of intermediate filaments in myocytes and epithelial cells^22, 23^. Plectin 1 also controls the formation of distinct circular focal adhesions at the perinuclear membrane of alveolar epithelial cells in response to the lung’s mechanical environment^72^, and regulates cell-matrix interaction and signaling through the mechano-receptor dystroglycan^73^. Taken together, IAV infection through B2 could disrupt mechanosensitive responses and remodeling of extracellular matrix causing irreparable structural injury and inflammation.

Collectively, the present findings support the model that IAV co-opts endophilin B2 in lung host cells to dysregulate host protective respiratory gene regulation and respiratory tissue remodeling resulting in respiratory dysfunction and immune cell dysregulation by IAV that impairs recovery from severe influenza infection. Our findings highlight endophilin B2 as a therapeutic target to alleviate influenza virus induced ARDS.

## Electronic supplementary material

Supplementary Figures
