# Supplementary material for: SH3GLB2/endophilin B2 regulates lung homeostasis and recovery from severe influenza A virus infection
Source: Sci Rep. 2017 Aug 4;7:7262. doi: 10.1038/s41598-017-07724-5 (PMC5544693; doi:10.1038/s41598-017-07724-5)
Supplement: Supplementary file 1 — Supplementary Figures [file 41598_2017_7724_MOESM1_ESM.pdf]

SUPPLEMENTARY FIGURES

Title:

SH3GLB2/endophilin B2 regulates lung homeostasis and recovery from severe influenza A virus infection

List of Authors:

Kristin K Fino, Linlin Yang, Patricia Silveyra, Sanmei Hu, Todd M. Umstead, Susan Diangelo, E. Scott Halstead, Timothy K Cooper, Thomas Abraham, Yoshinori Takahashi, Zhixiang Zhou, Hong Gang Wang, and Zissis C. Chroneos

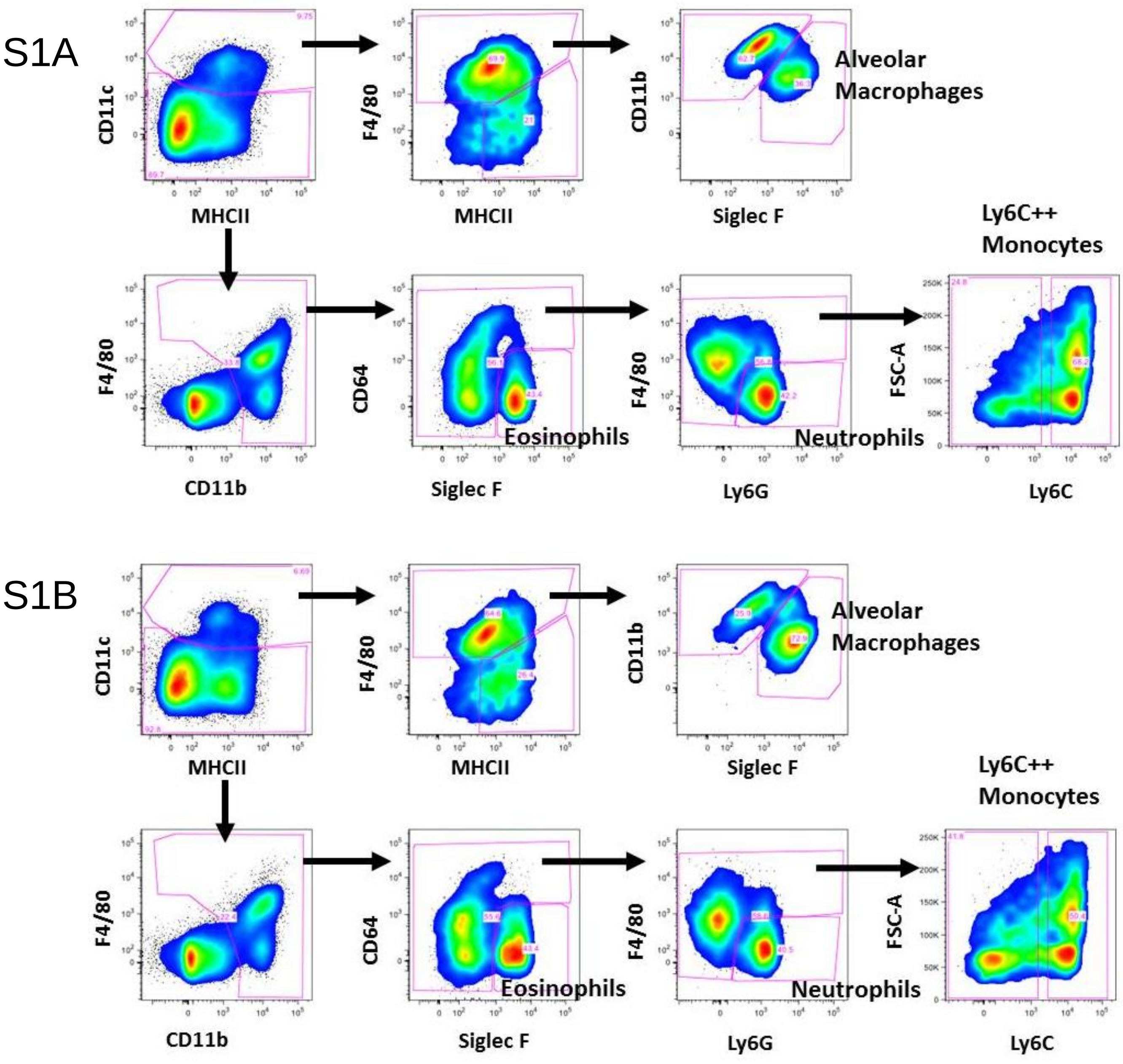

Supplementary Figure S1: Flow cytometry gating strategy. Representative flow cytometry profiling for lung cells obtained on day 12 after infection of WT (S1A) and B2-/- (S1B) mice.

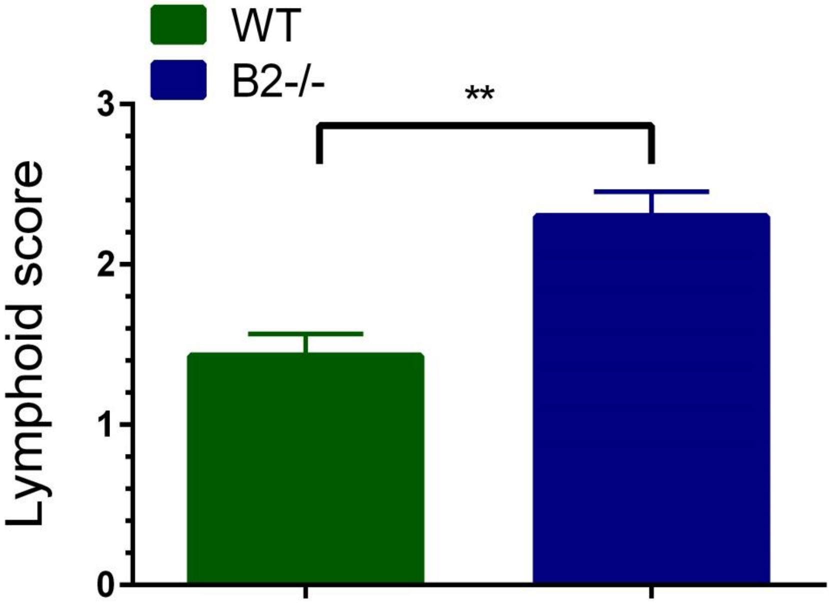

Supplementary Figure S2: **Peribronchiolar lymphoid cuffing sores**. Lung tissues sections from WT and B2-/- mice stained with H&E were scored for peribronchiolar lymphocytic cuffing as described in “Materials and Methods”. Data shown are means±SEM from tissue section of n=7 WT mice and n=5 B2-/- mice. **p<0.0004.

S3A

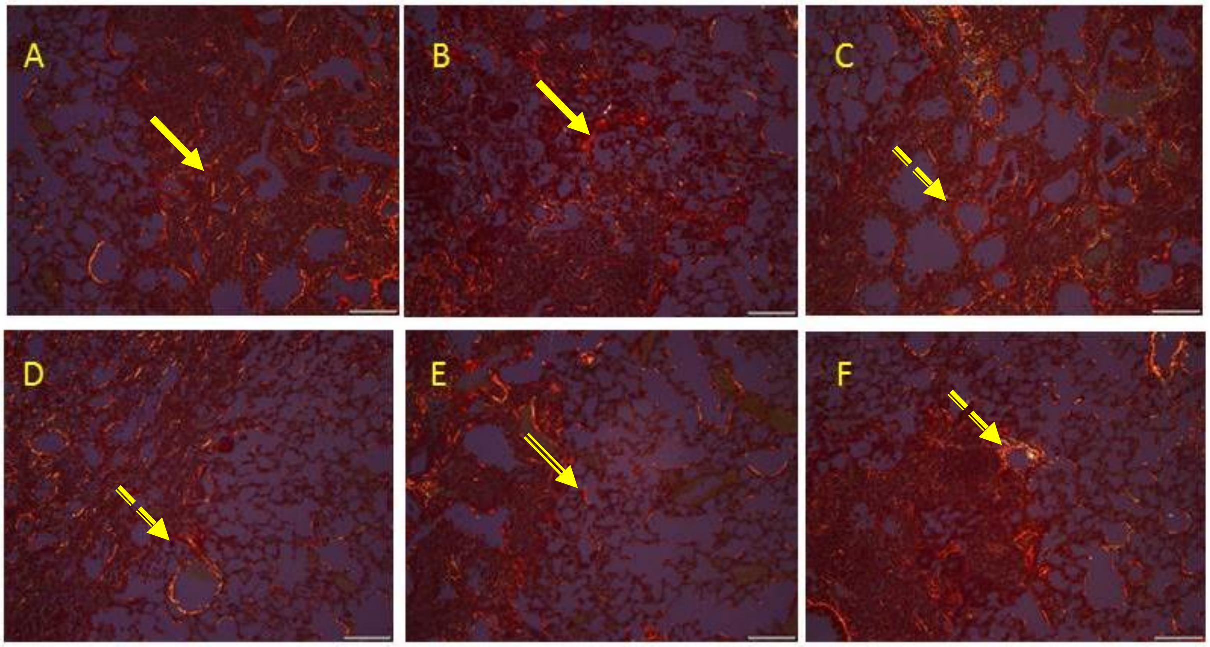

S3B

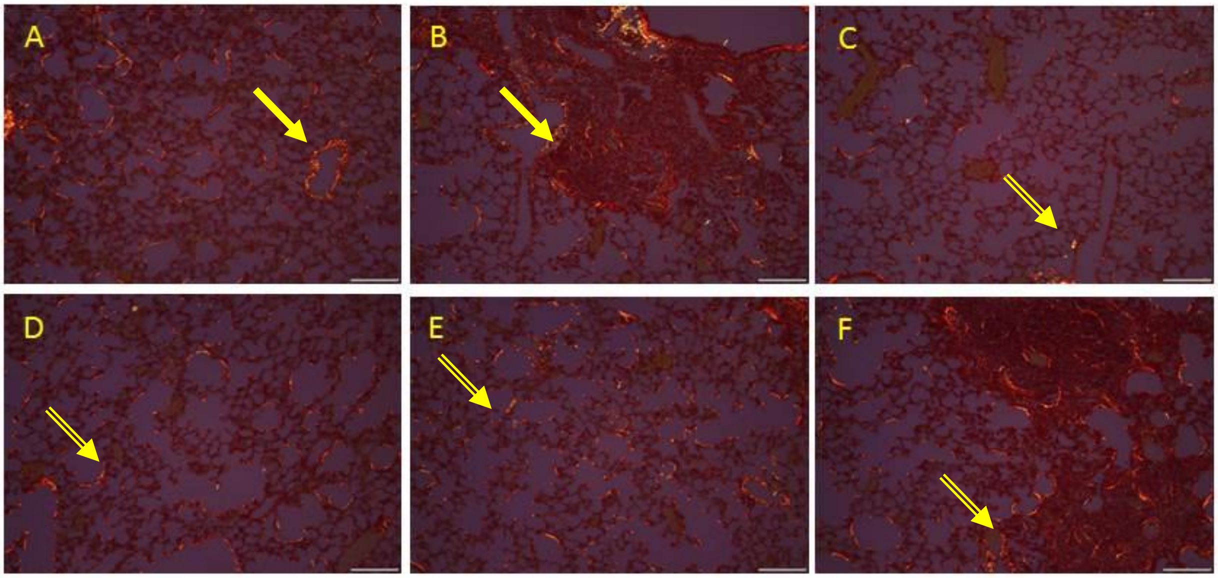

Supplementary Figure S3: Representative picric acid stained lung tissue sections from day 12 IAV infected lungs from WT (S2A) and B2-/- (S2B) mice. Picrosirius red stained sections visualized by optical birefringence microscopy were used for quantitative analysis of collagen birefringence light intensity derived from alveolar (lined arrows), bronchiolar/vascular (broken lined arrows), and in intralaveolar lesions (solid arrows) as described in “Materials and Methods”.

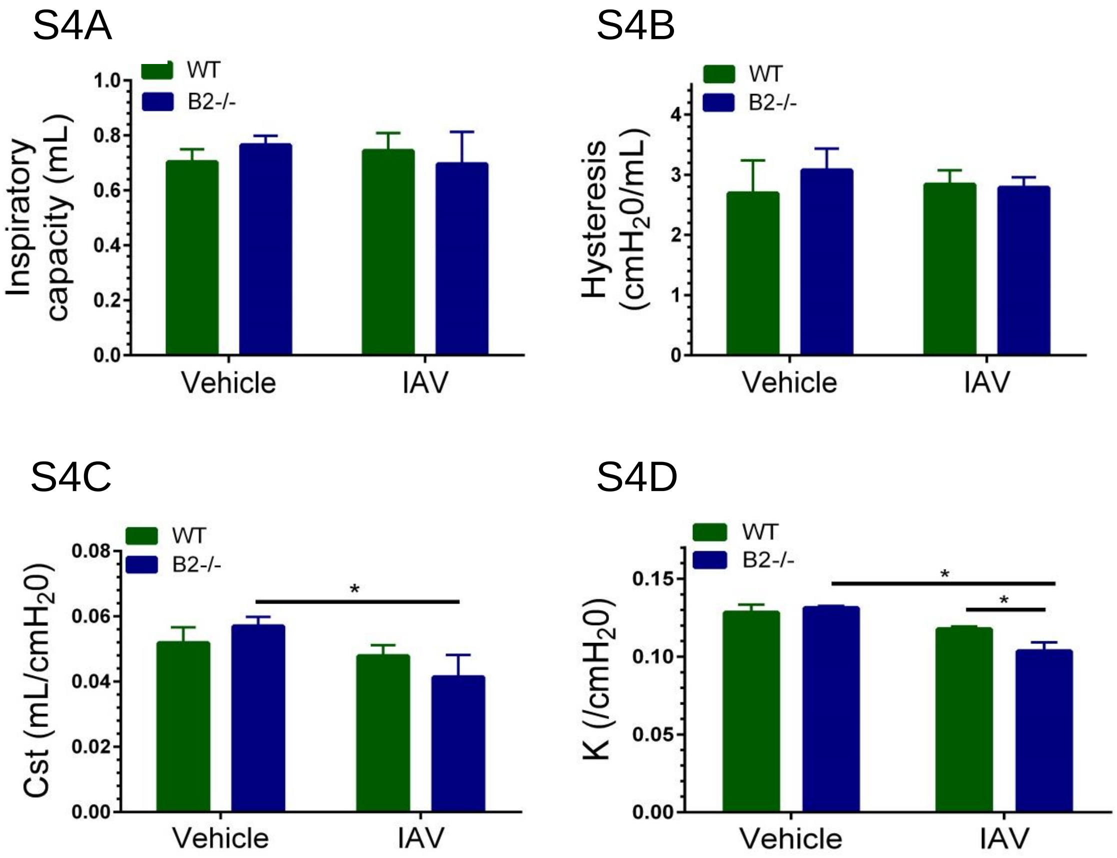

Supplementary Figure S4. Respiratory mechanical measurements in WT and B2-/- mice before and after IAV infection. The inspiratory capacity (S3A), hysteresis (S3B), quasi-static compliance (S3C), and curvature parameter (S3D) were calculated by fitting the Salazar-Knowles equation to the expiratory branch pressure-volume curves generated using a FlexiVent ventilator[1] as described in “Materials and Methods” in WT and B2-/- mice 14 days after administration of vehicle or IAV. Data shown are means±SD, n=4 mice per group. In S3C, *p<0.022 between vehicle and IAV treatment in B2-/- mice. In S3D, *p<0.027 between WT and B2-/- treatment groups, and *p<0.0011 between vehicle and IAV treatment in WT.

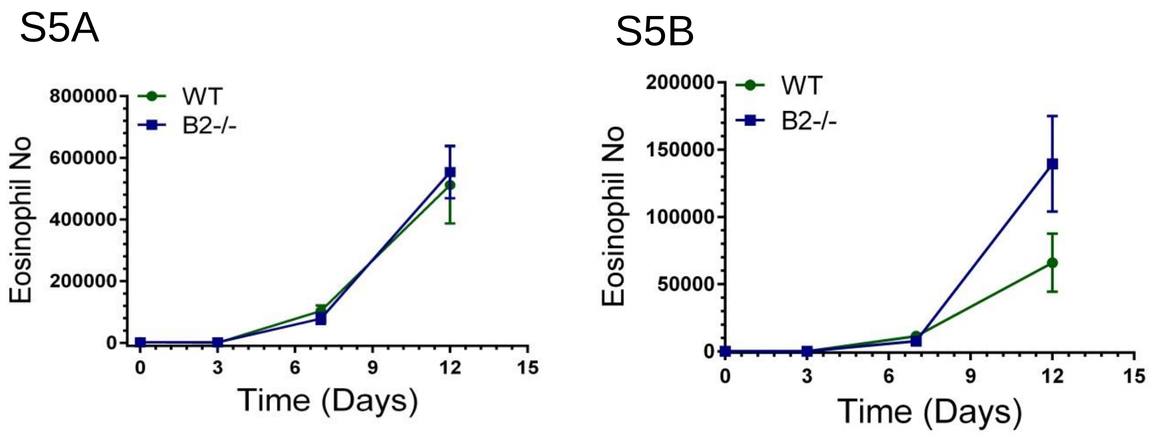

Supplementary Figure S5. Similar eosinophil recruitment was measured in response to IAV infection in WT and B2 mice. (S2A) Number of eosinophils in lungs. (S2B) Number of eosinophils in BAL.

1 McGovern, T. K., Robichaud, A., Fereydoonzad, L., Schuessler, T. F. & Martin, J. G. Evaluation of respiratory system mechanics in mice using the forced oscillation technique. *Journal of visualized experiments : JoVE*, e50172, doi:10.3791/50172 (2013).

SUPPLEMENTARY WESTERN BLOT IMAGES

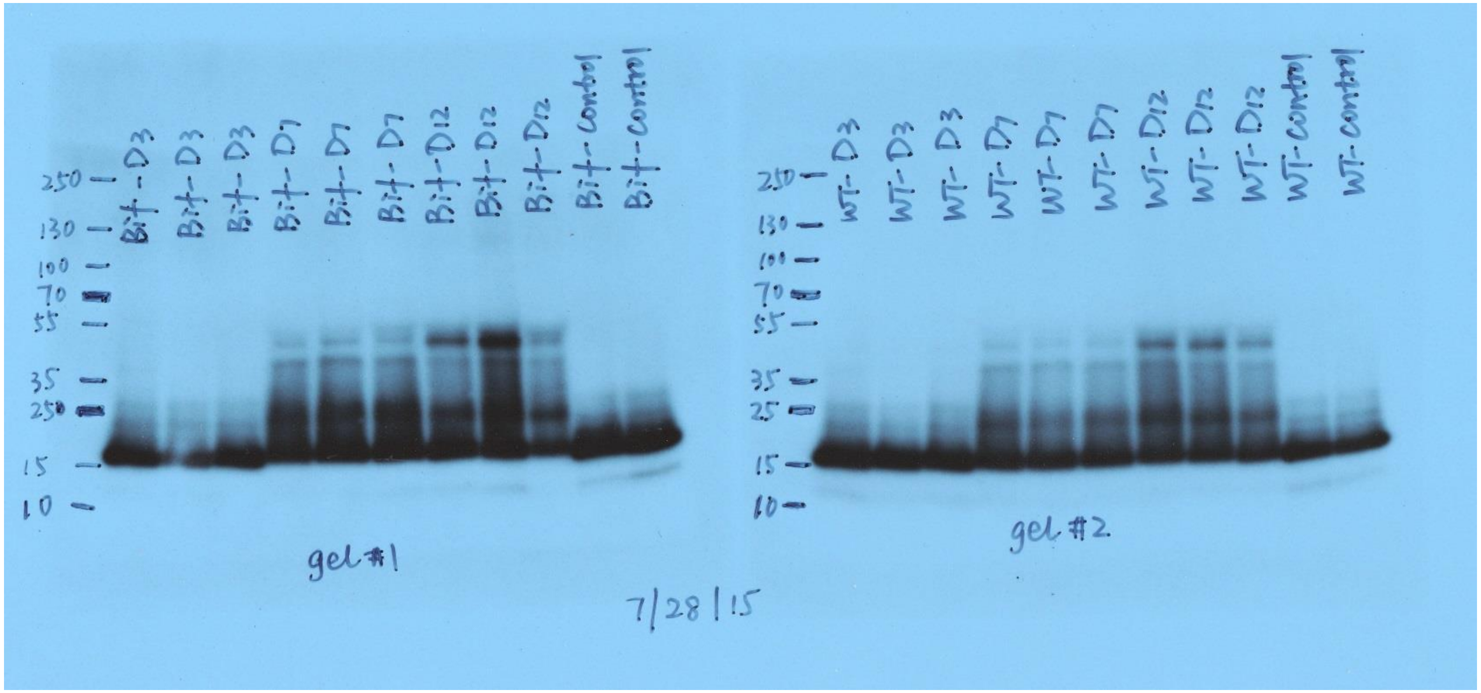

Non-reducing gel probed with anti-SP-B antibody

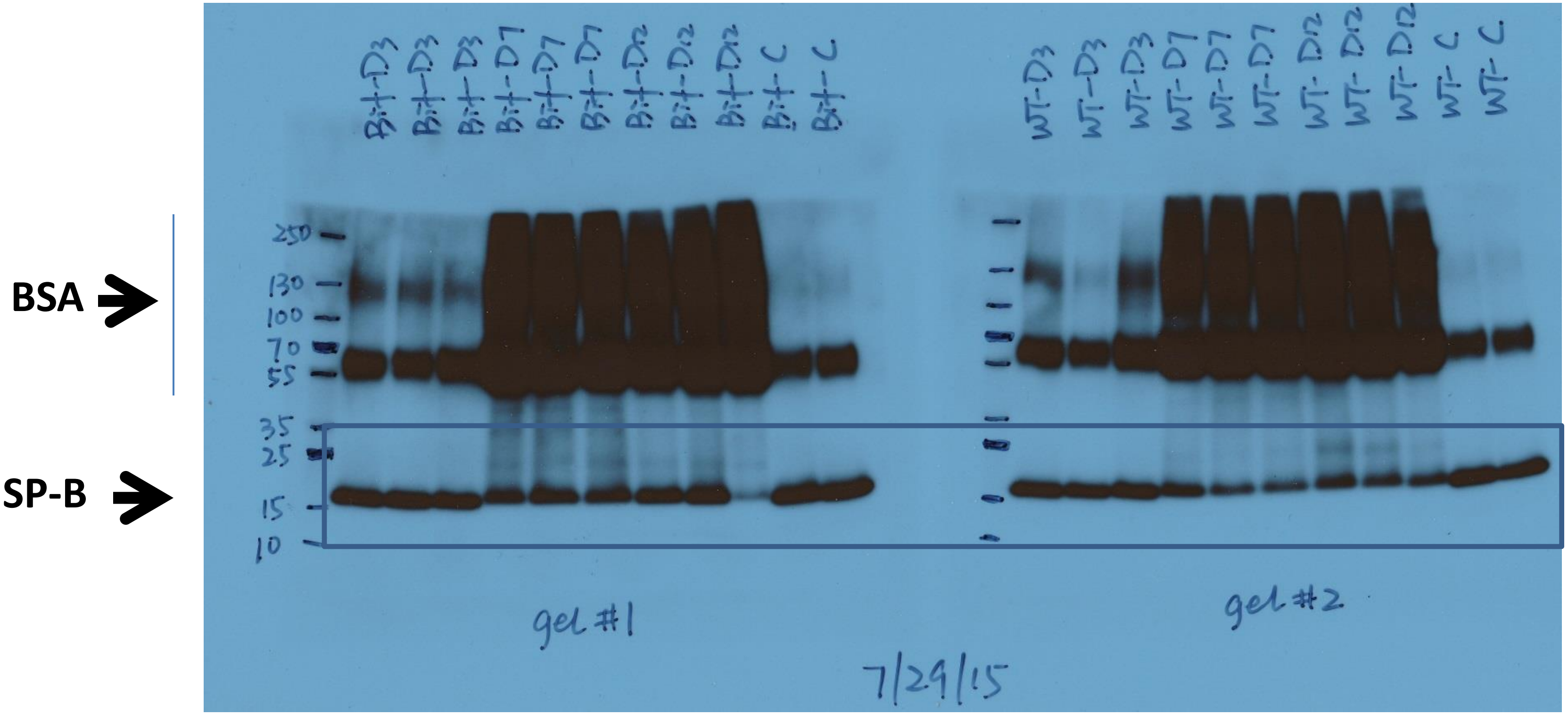

Non-reducing gel probed sequentially with anti-SP-B and anti BSA anti-bodies
The blots shown in Figure 3A were cropped from this blot in the boxed enclosure

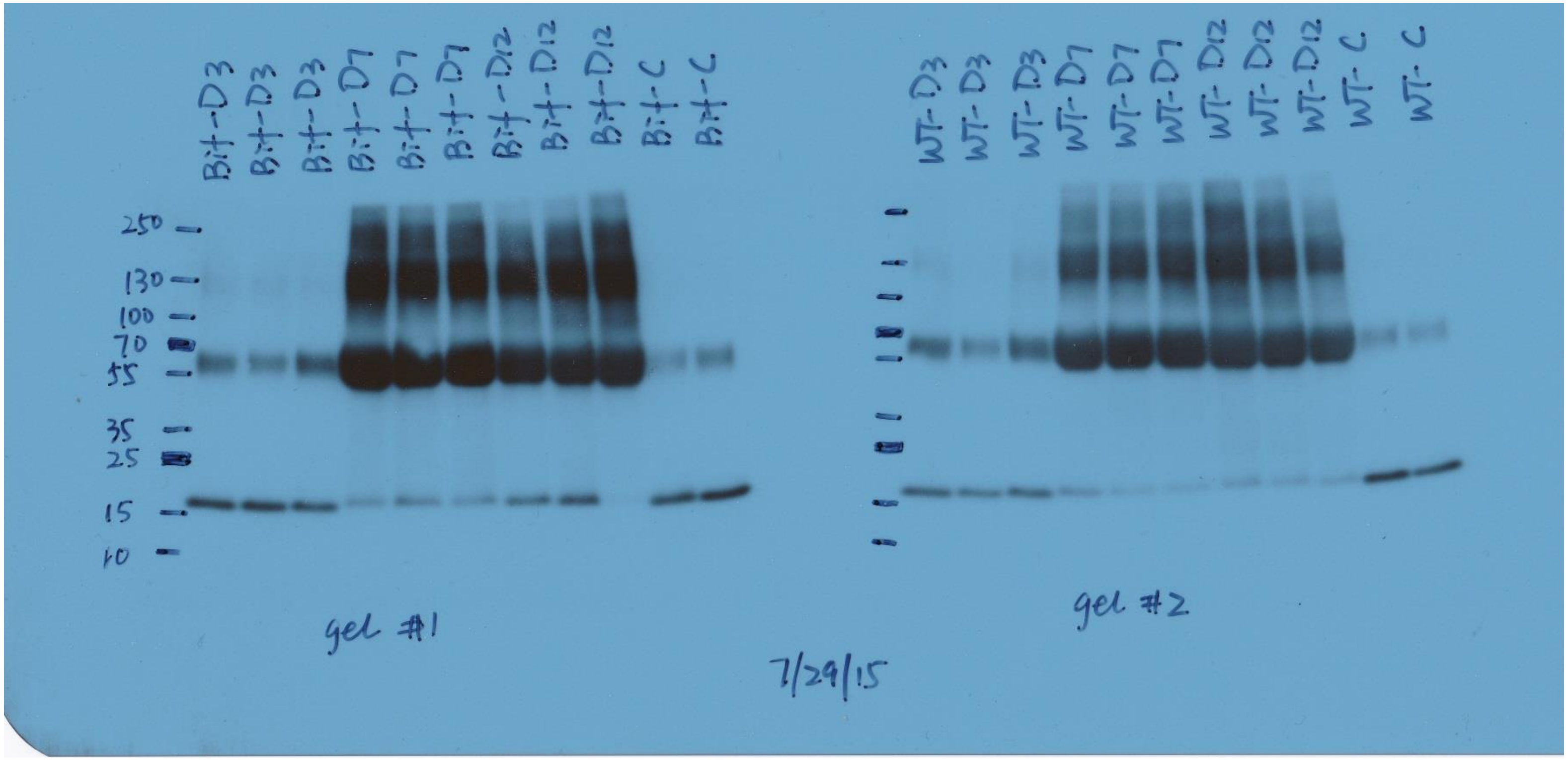

A shorter exposure of the above non-reducing gel probed sequentially with anti-SP-B and anti BSA anti-bodies

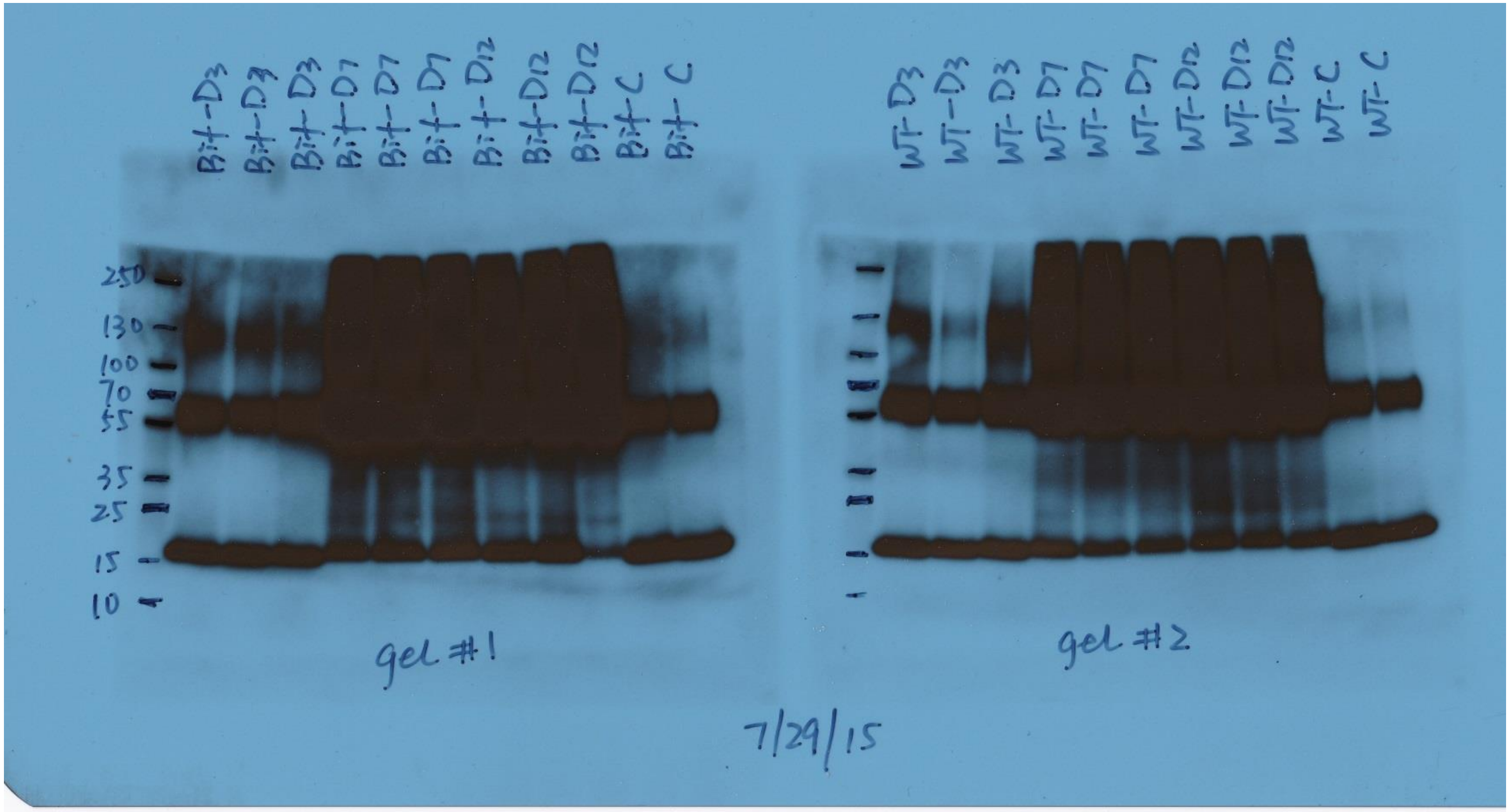

A longer exposure of the above non-reducing gel probed sequentially with anti-SP-B and anti BSA anti-bodies
